# Characterization of a Novel Bacteriophage swi2 Harboring Two Lysins Can Naturally Lyse *Escherichia coli*

**DOI:** 10.3389/fmicb.2021.670799

**Published:** 2021-05-25

**Authors:** Bingrui Sui, Xin Qi, Xiaoxue Wang, Huiying Ren, Wenhua Liu, Can Zhang

**Affiliations:** College of Veterinary Medicine, Qingdao Agricultural University, Shandong, China

**Keywords:** bacteriophage swi2, biological and genomic character, endolysin, holing, antibacterial activity

## Abstract

The novel virulent *Siphoviridae* bacteriophage swi2 was isolated from a pig farm, and its biological characteristics, genome architecture, and infection-related properties were characterized. Phage swi2 has a high titer of 1.01 × 10^12^ PFU/mL with good tolerance to UV rays and remains stable in the pH range of 6–10 and at temperatures less than 50°C. One-step growth analysis revealed that phage swi2 had a 25 min latent period with a large burst size (1,000 PFU/cell). The biological characteristics indicated that swi2 had good host infectivity and effective lytic activities. The genome of phage swi2 is composed of 47,611 bp with a G + C content of 46.50%. Eighty-nine orfs were predicted, and only 18 of them have known functions. No virulence genes or drug resistance genes were found in the genome. Genome sequence comparison of phage swi2 showed that there were a total of 10 homologous phages in the database with low similarity (less than 92.51% nucleotide identity and 66% query coverage). The predicted host lysis-related genes of phage swi2 consist of one *holin*, two *endolysins*, and *Rz/Rz1* equivalents. Antibacterial activity assays showed that both endolysins could naturally reduce the host *Escherichia coli* 51 titers by -1 log unit both *in vitro* and *in vivo*, EDTA showed no obvious synergistic action, and holin had no lytic effects on the host cell. These results provide necessary information for the development of antibiotic alternatives for the treatment of multidrug-resistant *Escherichia coli* infection.

## Introduction

*Escherichia coli* (*E. coli*) is widely distributed in the environment as one of the most important opportunistic pathogens and can cause colibacillosis of swine, which seriously threatens animal health globally, especially in high-density breeding farms (Tran et al., 2018). *E. coli* mainly infects 1–2-week-old piglets and causes white scour, yellow scour or porcine edema disease with clinical symptoms of diarrhea, which leads to very large economic losses (Sun et al., 2019). Antibiotics are currently used to treat colibacillosis in piglets. However, the development of antibiotics can hardly keep up with the rapid emergence of antagonistic bacteria in recent years (Lee et al., 2018). Therefore, new antibacterial agents have been encouraged for the control of pathogens, and bacteriophages or their endolysins are among the most promising candidates (Guenther et al., 2017). It has been reported that phages are the most abundant and diverse life forms on earth, and although some of them have been sequenced and reported, there is still much work to do in the process of phage therapy. In the past decade, phages and their enzymes have become a hot topic in the field of developing antibiotic substitutes, and there have been many successful phage therapy reports regarding the prevention and treatment of bacterial infections, suggesting that phages are good candidates as antibiotic alternatives (Vahedi et al., 2018). A bacteriophage (phage) with a broad host range, efficient proliferation, a high lysis titer and stable physical is more dominant in the prevention and control of colibacillosis. In this way, it can avoid the failure of prevention and control in the process of phage preparation to prevent and control bacterial infections based on the mismatch between the lysis range and the host bacteria (Alemayehu et al., 2012). Endolysin (lysin), a phage enzyme, has a broader lysis spectrum than its original phage. To date, most of the reported endolysins have shown good bactericidal activity against gram-positive bacteria but have shown less activity against gram-negative bacteria due to the outer membrane barrier, and the endolysins generally require the help of membrane permeating agent (Young, 2013). Recently, limited lysins have been reported that show bactericidal activity against gram-negative bacteria, it showed that the ability of lysins to pass bacterial outer membrane is mediated by the naturally occurring sequence of two ends (residues 1–20) carrying positively charged or hydrophobic residues (Plotka et al., 2019; Lai et al., 2020; Wang et al., 2020).

In the current study, an enterophage was isolated from a pig farm which showed a very high titer. Its biological characteristics, genomic architecture and infection-related properties were analyzed and found it was a new phage with a good tolerance to physical and chemical factors with a large burst size and a short incubation period. Interestingly, the proteins encoded by two functional *lysin* genes of phage swi2 had natural antibacterial activity in gram-negative bacteria. This special ability of phage swi2 and its lysins makes the clinical treatment of pathogenetic and drug-resistant *E. coli* possible.

## Materials and Methods

### Samples and Bacteria Strains

Samples of sewage were collected from pig farms in Shandong, China. A total of 79 *E. coli* clinical isolates with different resistances to antibiotic were selected as strains for host range testin this study (Supplementary Table 1). All *E. coli* strains were cultured in Luria-Bertani (LB) culture at 37°C overnight and stored at 4°C until used.

### Phage Isolation

The *E. coli* 51 strain was used as the host cell in this study for phage isolation. Sewage samples were performed using a standard enrichment method to propagate the phages, and the phages were isolated using the conventional double-layer agar plate method as described before (Zhang et al., 2013). Briefly, the sewage sample was cultured with 500 μL of *E. coli* 51 in 50 mL of LB at 37°C overnight. Then, the mixture was centrifuged at 12,000 × *g* for 30 min to collect the supernatant, and the supernatant was filtered through a 0.22 μm filter. One hundred microliters of supernatant and 100 μL of *E. coli* 51 were mixed and incubated for 5 min at room temperature, added to 3 mL of melted 0.7% soft agar, plated on an LB agar plate, and cultured at 37°C for 6 h. A single plaque was selected and purified four times, and the purified phage was stored at -80°C in 30% glycerol until use.

### Electron Microscopy of Phage swi2

For morphological observation, 10 μL of purified phage suspension (10^10^ PFU/mL) was dropped onto a carbon-coated grid, negatively stained with 2% uranyl acetate for 5 min and observed by transmission electron microscopy (HT7700, Hitachi, Japan) at an accelerating voltage of 80 kV. The size of the phage particles was determined from at least 10 measurements.

### Host Range and EOP Analysis

The host range of phage swi2 was determined by a spot test with 79 clinical isolates. In brief, 5 μL of purified phage swi2 was spotted onto freshly seeded lawns of each bacterial strain, left to dry, and then incubated at 37°C for 6 h. Additionally, the efficiency of plating (EOP) was calculated by the spot test as described in a previous report (Chen et al., 2013). Briefly, 10 μL of different dilutions (10^10^–10^13^) of phage swi2 were spotted onto freshly seeded lawns of the clinical isolates and incubated for 6 h at 37°C. The EOP values were determined by calculating the ratio of PFUs of each phage-susceptible strain to the PFUs obtained with *E. coli* 51. Moreover, the phage swi2 titer was determined by *SYBR Green I-*based quantitative PCR. Briefly, *lysin1-SYBR*-specific primers for the real-time quantitative PCR system were designed (Supplementary Table 2), amplification was performed according to the kit’s instructions. And the recombinant plasmid pColdTF-lysin1-SYBR was constructed after correct identification. The copy number of the recombinant plasmid was determined, and the plasmid was diluted by 10 times ratio to obtain the standard substance and establish the standard curve, then the titer of phage swi2 was tested. The experiments were repeated three times.

### Stability of Phage swi2

The thermal stability, ultraviolet light, and pH sensitivity of phage swi2 were determined as previously reported (Jamal et al., 2015). In brief, for thermal stability tests, aliquots of phage swi2 were incubated at 40, 50, 60, 70, and 80°C for 1 h, and samples were taken at 20 min intervals. For the ultraviolet stability tests, aliquots of phage swi2 were incubated at 37°C (at a distance of 40 cm away from a UV lamp) for 1 h, and samples were taken at 10 min intervals. For the pH stability tests, aliquots of phage swi2 were incubated at 37°C in the pH range from 1–14 for 1–3 h, and samples were taken at 1 h intervals. The phage titer of each sample was determined by the double-layer agar plate method. Each assay was repeated three times.

### MOI Assay of Phage swi2

The multiplicity of infection (MOI) refers to the ratio of phage to host bacteria during the processes for infection. The host cell *E. coli* 51 was selected and cultured to the logarithmic growth phase, diluted to 10^7^ CFU/mL, and then coincubated with phage swi2 at different MOIs (0.001, 0.01, 0.1, 1, 10, 100, and 1,000) for 4 h at 37°C. An *E. coli* 51 culture without phage swi2 was used as the control. The phage titers of swi2 were tested by the double-layer agar plate method, and the most optimal MOI was determined.

### One-Step Growth of Phage swi2

The latent period and burst size of phage swi2 were determined by one-step growth analysis as described previously (Yang et al., 2019). In brief, the host bacterial strain (10^9^ CFU) and phage swi2 suspension (10^10^ PFU) were mixed at an MOI of 10, incubated at 37°C for 5 min, and then centrifuged at 13,000 × *g* for 30 s to remove the unabsorbed phage particles. The precipitate was washed with LB twice and resuspended in 7 mL of LB, followed by further incubation at 37°C for 4 h. Samples were collected every 5 min in the first hour, every 10 min in the second hour, and every 30 min in the last 2 h. The phage titer was determined by the double-layer agar plate method. The experiment was repeated three times.

### Sequencing and Genomic Analysis of Phage swi2

The genome of phage swi2 was extracted with the TIANamp Virus DNA/RNA kit (Tiangen Biotech Beijing Co., Ltd.) and sequenced on an Illumina HiSeq platform. Contigs were assembled using the *de novo* assembly algorithm Newbler version 3.0 with default parameters (Bao et al., 2014). Open Reading Frames (*orfs*) were predicted using GeneMark^1^ and the RAST website^2^ (Park et al., 2012). Potential tRNA genes were identified with tRNA-scan-SE^3^ (Lowe and Chan, 2016). Resistance genes and virulence genes were identified using the Antibiotic Resistance Genes Database^4^ and Comprehensive Antibiotic Resistance Database^5^. The transmembrane area of the *holin* gene was predicted by TMHMM^6^. The domain of *lysin* genes was analyzed by CD-search in NCBI^7^. Phylogenetic analyses of phage swi2 were performed in the NCBI GenBank database based on the genome sequence or large terminase subunit sequences, and phylogenetic trees were constructed using the neighbor-joining method with the default parameters in MEGA 7.0 software (Kumar et al., 2016). The genome sequence of swi2 was deposited in GenBank under accession number MT768060.

### Antibacterial Activity Assay of Lytic System-Associated Proteins

Predicted *lysin* genes *orf 50* (*lysin1*), *orf 51* (*lysin2*), and *holin* gene (*orf 49*) were amplified from extracted phage swi2 genomic DNA by PCR using the designed primers (Supplementary Table 2) to construct recombined plasmids pColdTF-holin, pColdTF-lysin1, and pColdTF-lysin2, and plasmids were transferred into *E. coli* BL21 to express proteins. One hundred microliters of *E. coli* BL21 carrying the recombinant plasmid was cultured to the logarithmic growth period in LB broth at 37°C and induced with 1 mM IPTG at 16°C for 12 h. Then, the cultures were processed and sonicated to collect expressed proteins as previously reported (Zhang et al., 2015). The soluble expressed proteins were purified by Ni-affinity chromatography (CoWin Biosciences). The antibacterial activities of the proteins lysin1, lysin2, and holin against *E. coli* BL21 were tested both *in vivo* and *in vitro*. The antibacterial activities were evaluated by bacterial turbidity changes and bacterial log reductions. Briefly, for the antibacterial activity assay *in vivo*, *E. coli* BL21 carrying different plasmids was cultured to the logarithmic stage in LB at 37°C and induced with 1 mM IPTG for 16 h. The absorbance of the cultures at OD_600_ was measured at 0, 4, and 16 h in a 96-well plate. Additionally, colony counts of the cultures were carried out at 16 h. *E. coli* BL21 carried different plasmids without IPTG induction, and *E. coli* BL21 carrying the pColdTF plasmid with or without IPTG induction was used as a control. For the antibacterial activity assay *in vitro*, 100 μL of purified proteins (2 μM final concentration) was mixed with 100 μL of *E. coli* BL21 (10^6^ CFU/mL) in a 96-well plate with or without EDTA (2 μM final concentration) and incubated at 37°C for 4 h. The absorbance at OD_600_ was measured each hour and colony counts were carried out after 4 h. Trigger factor (TF) protein expressed by the pColdTF plasmid was used as the control. All the experiments were repeated three times.

## Results

### Morphological Characterization of Phage swi2

**FIGURE 1:**
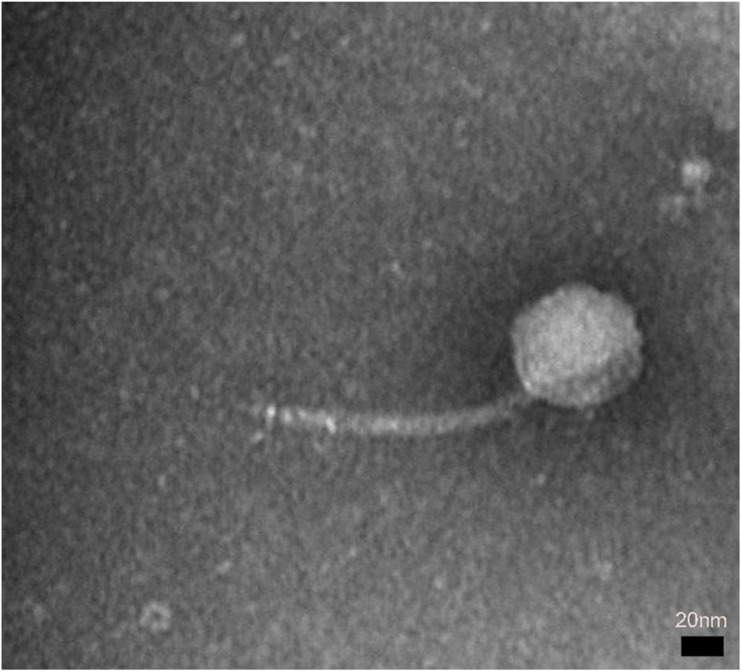
The morphology of phage swi2. Phage swi2 was negatively stained with 2% uranyl acetate and observed by transmission electron microscopy (TEM) at an accelerating voltage of 80 kV. The scale bars represent 20 nm.

A novel phage, swi2, targeting *E. coli* was isolated from a pig farm. The phage swi2 was observed under transmission electron microscopy (HT7700, Hitachi, Japan), which showed that it had a regular icosahedral head (80 nm in diameter) and an inextensible tail 140 nm in length (Figure 1). According to the current guidelines of the International Committee on Taxonomy of Viruses (ICTV), phage swi2 belongs to the family *Siphoviridae*, order *Caudovirales*.

### Biological Characterization of Phage swi2

**FIGURE 2:**
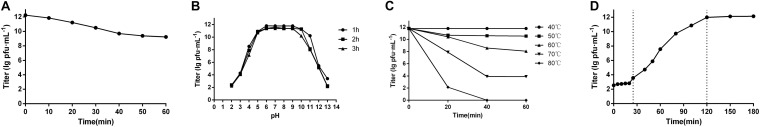
Biological characteristics of phage swi2. **(A)** UV stability, **(B)** pH stability, **(C)** thermal stability, and **(D)** one-step growth curve. Data are expressed as the means. Phage swi2 showed good physical and chemical tolerance, and had a very high titer and burst size in *E. coli 51* with a short latent period.

The *E. coli* 51 strain was selected as the host cell to test the biological characterization of phage swi2. Phage swi2, after propagation at an MOI of 10, showed a high titer of 1.01 × 10^12^ PFU/mL. Furthermore, the efficiency of plating (EOP) of swi2 was tested to confirm the extremely high titer, which showed a titer range from 1.7 × 10^10^ PFU/mL to 2.3 × 10^13^ PFU/mL in different host strains (Table 1). Quantitative fluorescent PCR was also used to test the phage swi2 titer, the results showed that the copy number of recombinant plasmid pColdTF-lysin1-SYBR was 6.15 × 10^13^ copies/mL and established the standard curve y = −3.086x + 42.852, then the titer of phage swi2 showed similar results (Supplementary Figure 1). In addition, phage swi2 tolerated UV light well (Figure 2A) and remained stable in the pH range of 6–10 (Figure 2B) and at a temperature less than 50°C for at least 1 h (Figure 2C). A one-step growth test showed that the latent period of swi2 was approximately 25 min, after which there was a rapid increase in the number of released viral particles until approximately 95 min, and the burst size was as high as 1,000 PFU/cell (Figure 2D).

**TABLE 1:** The titer and EOP of phage swi2 in host bacteria.

Strain numbers	Strain name	Titer phage swi2 (PFU/mL)	The efficiency of plating (EOP)
1	*E. coli* 21	1.7 × 10^10^	0.02
2	*E. coli* 24	1.4 × 10^12^	1.39
3	*E. coli* 30	2.3 × 10^13^	22.77
4	*E. coli* 38	1.4 × 10^13^	13.86
5	*E. coli* 42	1.3 × 10^13^	12.87
6	*E. coli* 45	1.1 × 10^13^	10.89
7	*E. coli* 46	1.1 × 10^12^	1.09
8	*E. coli* 48	8.0 × 10^11^	0.79
9	*E. coli* 49	1.0 × 10^11^	0.10
10	*E. coli* 51	1.01 × 10^12^	1.00
11	*E. coli* 54	7.0 × 10^11^	0.69

### Genomic Characterization of Phage swi2

**FIGURE 3:**
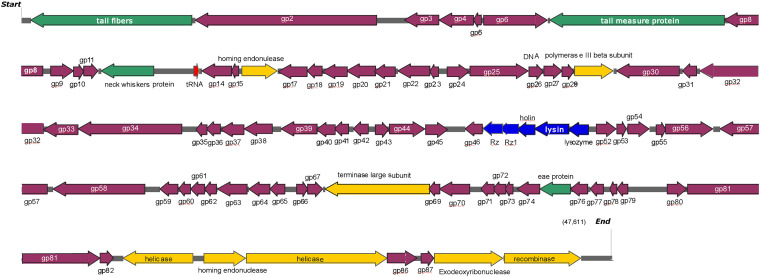
Detailed phage swi2 genome annotation. The arrow represents the direction of transcription. Different colors represent orfs with different predictive functions. The structural proteins are represented in green, the cleavage-related proteins are represented in blue, the proteins associated with transcription are represented in yellow, tRNA is represented in red and the hypothetical proteins are represented in purple.

The genome of phage swi2 was sequenced and analyzed. The phage swi2 genome contains 47,611 bp with a 46.50% G + C content. Eighty-nine *orfs* were predicted, 31 of which were positive-stranded, while the others were negative-stranded. Only 18 of 89 *orfs* were annotated as functional genes, including four structure-related genes, eight genes of transcription and replication, five lysis-related genes, and one *tRNA*^*Met*^ gene. No virulence genes or drug resistance genes were found in the genome. Detailed phage swi2 genome annotation showed that the *orfs* related to transcription and replication were mainly concentrated in the downstream part of the whole genome, while the structurally related *orfs* were scattered throughout the whole genome sequence (Figure 3).

The lysis cassette of swi2 was composed of the genes holin (*orf 49*), lysin1 *(orf 50)*, lysin2 (*orf 51*), and *Rz/Rz1* equivalents (*orf 47* and *orf 48*). *Rz/Rz1* equivalents consist of a type II integral membrane protein and an outer membrane lipoprotein, which can destroy the outer membrane after lysin destroys the peptidoglycan of the host cell (Summer et al., 2007; Catalao et al., 2013; Young, 2014). In this study, phage swi2 employed two components spanning *Rz* and *Rz1* and might play a role in the final step of gram-negative host lysis. *Orf 49*, downstream of the *Rz/Rz1* equivalents, was predicted as the *holin* gene and had 96.2% amino acid sequence identity with that of *Salmonella phage Skate* as found in the GenBank database. Structural analysis showed that there were two transmembrane domains and belonged to the type II holin (data not shown).

Most phages lyse bacteria through the holin-lysin mechanism. Lysin, expressed at the late stage of the phage, is essential for the release of progeny phages. Based on the enzymatic activity, lysins can be classified into five types (Oliveira et al., 2014). Two *lysin* genes (*orf 50* and *orf 51*) were predicted in phage swi2, and their enzymatic activities were analyzed. Lysin1 has the activity of glycoside hydrolase, and lysin2 has the activity of muraminidase, both of which hydrolyze β-1, four glycosidic bonds in murein.

Generally, lysins for gram-positive bacteria are modular with an N-terminal catalytic domain (CD) and a C-terminal cell wall binding domain (CBD). However, phages infecting gram-negative bacteria express lysins that are, in general, single domain structures with a few exceptions (Young, 2014). In this study, a CD search was used to analyze the conserved domains of lysin1 and lysin2, and no CD or CBD domain was found (data not shown).

### Phylogenetic and Genomic Analysis of Phage swi2

**FIGURE 4:**
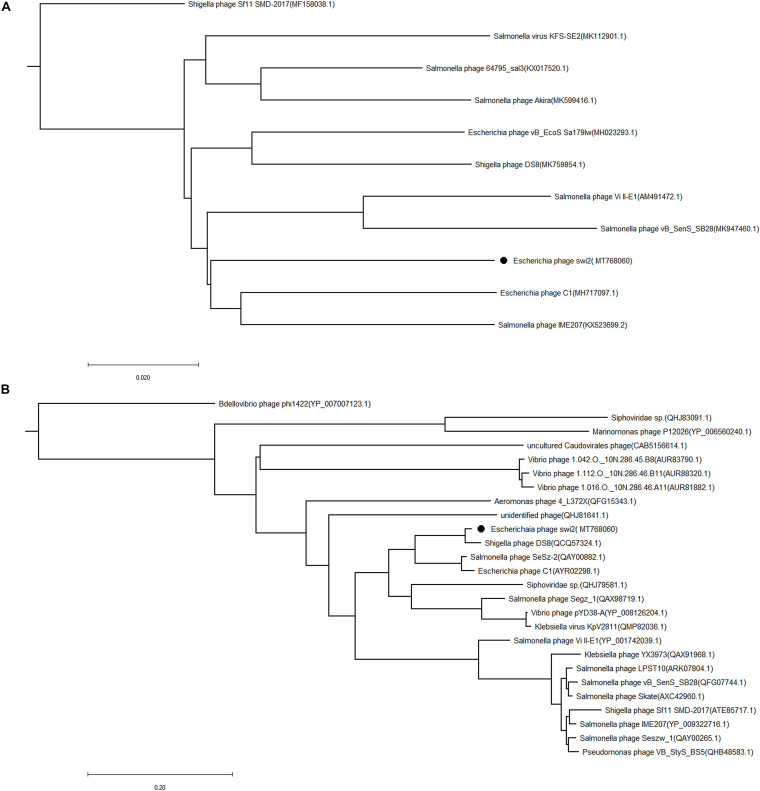
Phylogenetic analysis of the phage swi2. The sequences of **(A)** the whole genome and **(B)** the terminase large subunit were compared in the NCBI GenBank database, and the phylogenetic tree was generated using the neighbor-joining method with default parameters in MEGA 7.0. • Represents phage swi2.

The phylogenetic tree of phage swi2 was constructed based on the genomic sequence and amino acid sequence of the terminase large subunit (encoded by *orf 68*). The homology comparison results showed that the terminase large subunit is highly conserved in phages, which means it was a good candidate to determine the homology relationship. Based on the terminase large subunit, 25 homologous phages were classified into two groups (Figure 4B). The phage swi2 had the highest homology with *Shigella phage DS8* (QCQ57324.1) and was the farthest from *Shigella phage Bdellovibrio phage phi1422* (YP_007007123.1) of the other group. All of the homologous phages had no classification in genera degree.

Compared with the terminase large subunit, the phylogenetic tree based on the whole genomic sequence of phage swi2 showed a significant difference. There was low homology between swi2 and the phages registered in the NCBI database. A total of 10 phages in the database had homology with swi2. The highest relationship with phage swi2 was *Escherichia* phage C1 (AYR02298.1), with only a 66% coverage rate and 92.51% identity, followed by *Salmonella* phage IME207 (YP_009322716.1), with a 53% coverage rate and 92.10% identity. Phage swi2 had the most remote relationship with *Shigella phage Sf11 SMD-2017* (ATE85717.1) (Figure 4A). Similarly, all phages were not classified to genera. Phages with ≥40% protein homologs were grouped into the same genus (Wittmann et al., 2015). Therefore, we suggest that phage swi2 and 10 homologous phages in the database should be classified into a new genus.

### Expression and Antibacterial Activity of Lysis-Related Genes

**FIGURE 5:**
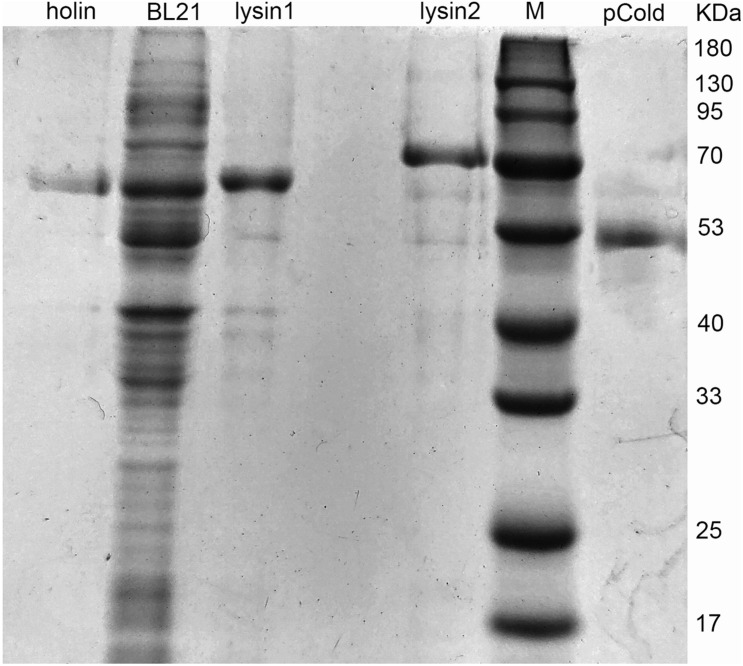
Protein expression of holin, lysin1, and lysin2. *E. coli* BL21 harboring the recombinant plasmids (pColdTF-holin, pColdTF-lysin1, pColdTF-lyisn2) were induced with 1 mM IPTG at 16°C for 16 h. After ultrasonic purification, the expressed proteins in the supernatant were collected and detected by SDS-PAGE.

**FIGURE 6:**
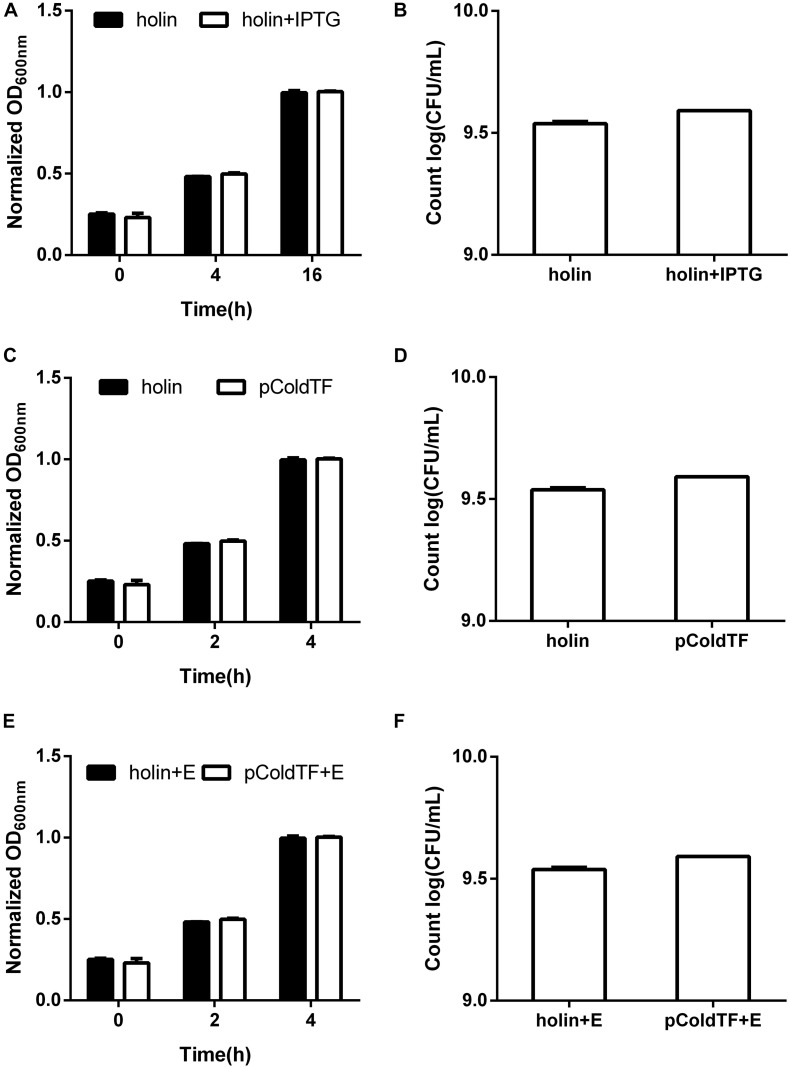
Lytic activity assay of the holin protein. *E. coli* BL21 carrying the pColdTF-holin plasmid was cultured in LB at 16°C. **(A)** The turbidity was detected at 0, 4, and 16 h with or without IPTG inducement, and **(B)** colony counts were performed at 16 h. The antibacterial activities of holin against *E. coli* BL21 were tested *in vitro* for the **(C)** absorbance at OD_600_ within 4 h and **(D)** colony count at 4 h. Similarly, the antibacterial activity of holin with EDTA against *E. coli* BL21 was determined by the **(E)** absorbance at OD_600_ within 4 h and **(F)** colony count at 4 h. The pColdTF protein with the same concentration was used as a control. Holin showed no lytic activity in any of the tests (*P* > 0.05).

*Holin*, *lysin1*, and *lysin2* were amplified by PCR to construct recombinant plasmids for expression in *E. coli* BL21. The expressed proteins were purified by affinity chromatography. SDS-PAGE results showed that the recombined proteins were all soluble and expressed in *E. coli* BL21 with masses of 61 kDa (holin), 69 kDa (lysin1), and 62 kDa (lysin2) (Figure 5). Holin had a low expression level and showed no antibacterial activity in both *in vitro* and *in vivo* tests (*P* > 0.05; Figure 6).

**FIGURE 7:**
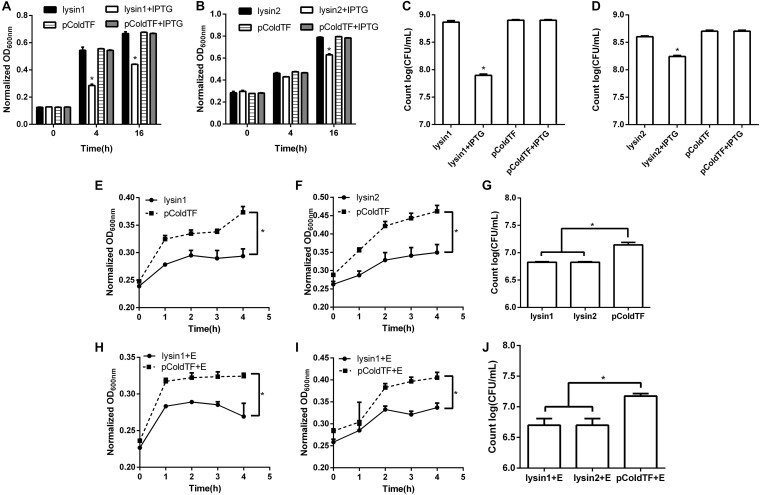
Lytic activity assay of lysin1 and lysin2. *E. coli* BL21 carrying the pColdTF-lysin1 or pColdTF-lysin2 plasmid was cultured in LB and induced with IPTG at 16°C to express proteins. The turbidities of **(A)** lysin1 and **(B)** lysin2 were detected by absorbance at OD_600_ at 0, 4, and 16 h, and colony counts were performed for **(C)** lysin1 and **(D)** lysin2 at 16 h. Lysin1 and lysin2 reduced titers by -1 log unit at 16 h and showed natural antimicrobial activities (0.01 < *P* < 0.05). The antibacterial activities against *E. coli* BL21 tested *in vitro* for the **(E)** lysin1 and **(F)** lysin2 absorbance at OD_600_ within 4 h; also, **(G)** *E. coli* BL21 colonies were counted at 4 h. Similarly, the antibacterial activities of lysin1 and lysin2 with EDTA on *E. coli* BL21 *in vitro* were determined by **(H)** lysin1 and **(I)** lysin2 absorbance at OD_600_ within 4 h; also, **(J)** *E. coli* BL21 colonies were counted at 4 h. The pColdTF protein was used as a control. Lysin1 and lysin2 reduced the titer of *E. coli* BL21 by -1 log unit at 4 h with or without EDTA and showed natural antimicrobial activity (0.01 < *P* < 0.05).

Lysin1 and lysin2 were also soluble when expressed in *E. coli* BL21. The activity measurement results *in vivo* showed that lysin1 or lysin2 expression in *E. coli* BL21 led to an -1 log titer reduction at 16 h and showed antibacterial activity (0.01 < *P* < 0.05). In an *in vitro* test, proteins lysin1 and lysin2 showed natural antibacterial activities against *E. coli* BL21 (0.01 < *P* < 0.05), and compared with the control group, lysin1 and lysin2 reduced the titer of *E. coli* BL21 by -1 log unit in 4 h (Figure 7). It has been reported that EDTA can penetrate the outer membrane of gram-negative bacteria to help lysins have better cleavage effects (Oliveira et al., 2014). In this study, the coeffect of lysin1 or lysin2 with EDTA was also tested. There was no significant difference between groups with or without EDTA. The results of the antibacterial activity assay showed that lysin1 and lysin2 of phage swi2 could naturally lyse gram-negative bacteria *in vitro* and *in vivo*.

## Discussion

Colibacillosis is an important infectious disease that endangers the world pig industry. Currently, the prevalence of drug-resistant bacteria makes *E. coli* infections difficult to control by antibiotics, and new alternatives are urgently needed to treat this crisis. Phages and their lysins showed great therapeutic potential due to their capacity to induce immediate lysis of the target bacterium with no effects on the normal flora structure (Sillankorva et al., 2011). In the last decade, there have been numerous reports of phages successfully preventing and treating pathogenic bacteria (Vieira et al., 2012; Zhang et al., 2013; Waters et al., 2017). In this study, a novel virulent enterophage, swi2, was isolated, and its genomic and biological characteristics were investigated.

Phage swi2 had good tolerance to physical and chemical factors, such as UV stability, pH stability, and temperature stability. The phage swi2 had a 10^12^ titer and 10^3^ burst size in *E. coli* 51 with a short incubation period, which indicated good host infectivity and effective lytic activities. One phage had different titers and burst sizes in different hosts for the protein-synthesizing machinery of the host bacteria. To further confirm this result, we determined the titer of phage swi2 in all susceptible host bacteria with a spot test and quantitative fluorescent PCR. The EOP results showed that phage swi2 had a generally high titer in most host bacterial strains (10^10^–10^13^ PFU). In general, the phage titer and burst size were associated with the incubation period and the proportion of protein synthesis machinery of the host cell (Choi et al., 2010). The larger the size of the host cell and the longer the incubation period of the phage tend to result in a larger burst size. For example, The phage SA has 30 min incubation period with 1,000 PFU/cell burst size; the phage MS2 has 45 min latent period with 2,000 PFU/cell burst size; the Avian-pathogenic *E. coli* phage has 24 h incubation period and 2.4 × 10^4^ PFU/mL burst size (Horiuchi and Adelberg, 1965; Hamza et al., 2016; Fakhouri et al., 2019). In this study, phage swi2 has a short incubation period of 25 min with a high to 1,000 PFU/cell burst size, which indicates that swi2 has potential development value for clinical application.

The homological relationship of phage swi2 was analyzed based on the genome sequence and the amino acid sequence of the terminase large subunit. The terminase large subunit showed high conservation in the phylogenetic tree, which was consistent with previous reports (Daudén et al., 2013). However, the phylogenetic tree based on the genomic sequence of phage swi2 showed low homology with all 10 phages registered in the NCBI database. None of these phages were characterized or classified, which showed that swi2 is a new phage and is different from that of previous reports.

Phage swi2 was found to have one *holin* gene and two *lysin* genes, and it was speculated that phage swi2 lyses host bacteria through the holin-lysin system. However, lytic activity tests showed that holin had no lytic activity, while both lysins had intracellular and extracellular lytic activities. This showed that holin in phage swi2 was not essential in the process of phages lysing host bacteria, and multiple lysins can participate in the lysis process at the same time, which is consistent with previous reports (Catalao et al., 2011). Additionally, *Rz/Rz1* equivalents may play a role in the process of releasing progeny, which could destroy the outer membrane after lysin destroys the peptidoglycan of the host cell. The plaque of phage swi2 is very small, which may also be related to the inactivated holin. Interestingly, two functional *lysin* genes were found in the phage swi2, and both lysozymes showed strong activity to naturally lyse gram-negative bacteria without EDTA. To date, there have been few reports of lysozymes with natural lytic activities on gram-negative bacteria, and most of them have a positively charged hydrophobic amino acid at the N-terminus (Vázquez et al., 2018; Wang et al., 2020). In this study, amino acid sequence analysis of lysin1 and lysin2 showed a similar character: there were hydrophobic and positively charged amino acids at the N-terminus (data not shown).

In conclusion, the novel virulent *Siphoviridae* phage swi2 isolated in this study produced a very high titer and burst size with a short incubation period, it had low homology with phages registered in the NCBI database, and its lysins could naturally lyse gram-negative bacteria. These results provide necessary information for the development of antibiotic alternatives for the treatment of multidrug-resistant *Escherichia coli* infection.

## Data Availability Statement

The datasets presented in this study can be found in online repositories. The names of the repository/repositories and accession number(s) can be found below: NCBI Genbank MT768059 and MT768060.

## Author Contributions

BS and XQ performed the experiments, analyzed the data, and wrote this manuscript. XW, HR, and WL performed the experiments. CZ designed the experiments and revised the manuscript. All authors contributed to the article and approved the submitted version.

## Conflict of Interest

The authors declare that the research was conducted in the absence of any commercial or financial relationships that could be construed as a potential conflict of interest.

## Footnotes

1 http://topaz.gatech.edu/GeneMark/

2 https://rast.nmpdr.org/

3 http://lowelab.ucsc.edu/tRNAscan-SE/

4 http://ardb.cbcb.umd.edu/

5 http://arpcard.mcmaster.ca

6 http://www.cbs.dtu.dk/services/TMHMM-2.0/

7 https://www.ncbi.nlm.nih.gov/Structure/cdd/wrpsb.cgi
