# Supplementary material for: Characterization of a Novel Bacteriophage swi2 Harboring Two Lysins Can Naturally Lyse Escherichia coli
Source: Front Microbiol. 2021 May 25;12:670799. doi: 10.3389/fmicb.2021.670799 (PMC8185280; doi:10.3389/fmicb.2021.670799)
Supplement: Supplementary file 1 [file Data_Sheet_1.PDF]

Table S1

Drug sensitivity and lytic activity of 79 *E. coli* strains

Strain number	Strain name	Susceptibility results								Lysis results
		PEN	DOX	FFC	ENR	CIP	CTX	NEO	CRO	
1	*E. coli* 1	R	I	S	R	S	S	R	S	-
2	*E. coli* 2	R	R	S	I	S	I	R	S	-
3	*E. coli* 3	R	R	S	I	I	S	R	S	-
4	*E. coli* 4	R	R	I	S	S	I	R	S	-
5	*E. coli* 5	R	R	I	I	S	S	R	S	-
6	*E. coli* 6	R	R	I	I	I	I	R	S	-
7	*E. coli* 7	R	R	I	I	S	S	R	S	-
8	*E. coli* 8	R	R	R	R	S	S	I	S	-
9	*E. coli* 9	R	R	S	I	I	I	R	S	-
10	*E. coli* 10	R	R	S	I	S	I	I	S	-
11	*E. coli* 11	R	R	I	R	R	S	I	S	-
12	*E. coli* 12	R	R	I	I	I	I	I	I	-
13	*E. coli* 13	R	R	R	I	S	R	I	S	-
14	*E. coli* 14	R	R	R	I	I	R	I	R	-
15	*E. coli* 15	R	R	R	I	I	S	I	S	-
16	*E. coli* 16	R	R	R	R	I	S	R	S	-
17	*E. coli* 17	R	R	R	R	R	S	I	S	-
18	*E. coli* 18	R	R	R	I	S	S	R	S	-
19	*E. coli* 19	R	R	R	R	I	S	R	S	-
20	*E. coli* 20	R	R	S	S	S	S	S	S	-
21	*E. coli* 21	R	R	S	R	R	I	R	S	+
22	*E. coli* 22	R	R	R	R	S	I	R	S	-
23	*E. coli* 23	R	R	R	R	R	I	R	R	-
24	*E. coli* 24	R	R	R	R	R	I	I	I	+
25	*E. coli* 25	R	R	R	R	R	R	R	R	-
26	*E. coli* 26	R	R	I	S	S	I	I	S	-
27	*E. coli* 27	R	R	R	I	I	I	I	S	-
28	*E. coli* 28	R	R	I	I	R	I	I	I	-
29	*E. coli* 29	R	R	R	R	R	I	I	I	-
30	*E. coli* 30	R	R	R	I	I	S	I	S	+
31	*E. coli* 31	R	R	R	R	R	S	R	I	-
32	*E. coli* 32	R	R	R	I	I	S	I	S	-
33	*E. coli* 33	R	R	R	R	R	I	I	I	-
34	*E. coli* 34	R	R	R	S	S	S	R	S	-
35	*E. coli* 35	R	R	R	R	R	R	I	S	-
36	*E. coli* 36	R	R	R	R	I	I	R	S	-
37	*E. coli* 37	R	R	I	R	R	I	I	I	-

38	*E. coli* 38	R	R	S	I	I	S	R	S	+
39	*E. coli* 39	R	I	I	S	S	I	R	S	-
40	*E. coli* 40	R	R	I	I	S	S	I	R	-
41	*E. coli* 41	R	R	R	I	I	I	R	S	-
42	*E. coli* 42	R	R	I	I	S	S	R	S	+
43	*E. coli* 43	R	R	R	R	S	S	I	I	+
44	*E. coli* 44	R	R	S	I	I	I	R	S	-
45	*E. coli* 45	R	R	S	I	S	I	I	S	-
46	*E. coli* 46	R	R	I	R	R	S	I	S	+
47	*E. coli* 47	R	R	I	I	I	I	R	I	-
48	*E. coli* 48	R	R	R	R	S	R	I	S	+
49	*E. coli* 49	R	R	R	I	I	R	I	R	+
50	*E. coli* 50	R	R	R	I	I	S	R	S	-
51	*E. coli* 51	R	R	S	R	I	S	I	S	+
52	*E. coli* 52	R	R	R	R	R	R	R	S	-
53	*E. coli* 53	R	R	R	I	S	S	I	I	-
54	*E. coli* 54	R	I	S	R	I	S	R	S	+
55	*E. coli* 55	R	R	S	S	S	S	R	R	-
56	*E. coli* 56	R	R	S	R	R	I	S	S	-
57	*E. coli* 57	R	R	R	R	S	R	S	S	-
58	*E. coli* 58	R	R	R	R	R	I	R	R	-
59	*E. coli* 59	R	R	I	R	R	I	I	I	-
60	*E. coli* 60	R	I	R	R	S	R	R	I	-
61	*E. coli* 61	R	R	I	S	S	I	I	I	-
62	*E. coli* 62	R	R	R	I	I	S	I	S	-
63	*E. coli* 63	R	R	I	I	R	I	I	I	-
64	*E. coli* 64	R	R	R	R	R	I	I	I	-
65	*E. coli* 65	R	R	R	I	I	S	I	S	-
66	*E. coli* 66	R	R	R	R	S	S	R	I	-
67	*E. coli* 67	R	I	S	I	I	S	I	S	-
68	*E. coli* 68	R	R	R	R	R	I	I	I	-
69	*E. coli* 69	R	R	R	S	S	S	S	S	-
70	*E. coli* 70	R	R	S	R	R	R	I	S	-
71	*E. coli* 71	R	R	R	R	I	I	R	S	-
72	*E. coli* 72	R	R	I	R	R	I	I	S	-
73	*E. coli* 73	R	R	R	I	I	R	I	R	-
74	*E. coli* 74	R	R	R	I	I	S	R	I	-
75	*E. coli* 75	R	I	R	R	I	S	I	S	-
76	*E. coli* 76	R	R	I	R	R	S	R	S	-
77	*E. coli* 77	R	R	R	I	S	S	I	R	-
78	*E. coli* 78	R	R	R	R	I	S	I	I	-
79	*E. coli* 79	R	R	R	R	S	I	R	S	-

Note: PEN, Penicillin; DOX, Doxycycline hydrochloride; FFC, Florfenicol; ENR, Enrofloxacin; CIP, Ciprofloxacin; CTX, Cefotaxime; NEO, Neomycin; CRO,

Ceftriaxone; R, resistance; I, intermediate; S, sensitive; +, lysis; -, no lysis.
