# Supplementary material for: Characterization of a Novel Bacteriophage swi2 Harboring Two Lysins Can Naturally Lyse Escherichia coli
Source: Front Microbiol. 2021 May 25;12:670799. doi: 10.3389/fmicb.2021.670799 (PMC8185280; doi:10.3389/fmicb.2021.670799)
Supplement: Supplementary file 2 [file Data_Sheet_2.PDF]

Table S2

Sequences of PCR primers used for phage swi2 gene cloning

Name	Primer sequences (5’-3’)	Reaction conditions (Tm)	Amplified fragment length (bp)
holin	F:ggtatcgaaggtaggcatatgATGAAAAAACTAAGCAACTGGCTG<br>R:agcagagattacctatctagaGCGAGAATTGACATCATTGCG	59°C	240
lysin1	F:ggtatcgaaggtaggcatatgATGGATATTAGCAAAAACATGAAGGC<br>R:agcagagattacctatctagaTGCCACAACTCCGCCAGC	59°C	465
lysin2	F:ggtatcgaaggtaggcatatgATGGAACTATCGCAACGCGG<br>R:agcagagattacctatctagaATATCCATACCAGTAAACCTCTTTTTCA	59°C	279
lysin1-SYBR	F: GGATAACGGGCGACAGAA<br>R: AGCGGCAGTGGATTTGAT	60°C	133

Note: F, forward primer; R, reverse primer; the underlined bases showed restriction enzyme sites (*Nde* I and *Xba* I).
