# Supplementary material for: Characterization of a Novel Bacteriophage swi2 Harboring Two Lysins Can Naturally Lyse Escherichia coli
Source: Front Microbiol. 2021 May 25;12:670799. doi: 10.3389/fmicb.2021.670799 (PMC8185280; doi:10.3389/fmicb.2021.670799)
Supplement: Supplementary file 3 [file Data_Sheet_3.PDF]

Fig. S1

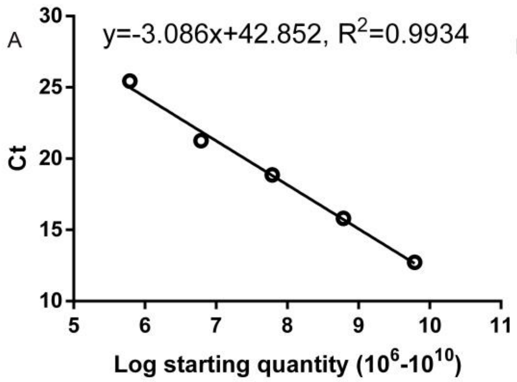

B

Copies	Ct
10^{10}	35.0268
10^9	32.0202
10^8	28.0221
10^7	25.5529
10^6	24.2997

(A) Standard curve of the recombinant plasmid pColdTF-lysin1-SYBR.

(B) The titer of phage swi2 was determined by quantitative fluorescent PCR.
