# Supplementary material for: Characterization of a Novel Bacteriophage swi2 Harboring Two Lysins Can Naturally Lyse Escherichia coli
Source: Front Microbiol. 2021 May 25;12:670799. doi: 10.3389/fmicb.2021.670799 (PMC8185280; doi:10.3389/fmicb.2021.670799)
Supplement: Supplementary file 4 [file Data_Sheet_4.PDF]

LOCUS MT768060 47611 bp DNA linear PHG 11-SEP-2020
DEFINITION Escherichia phage vB_EcoS_swi2, complete genome.
ACCESSION MT768060
VERSION MT768060
KEYWORDS .
SOURCE Escherichia phage vB_EcoS_swi2
ORGANISM Escherichia phage vB_EcoS_swi2
Viruses; unclassified viruses; unclassified bacterial viruses.
REFERENCE 1 (bases 1 to 47611)
AUTHORS Sui,B.
TITLE Complete genome sequence analysis of a novel Escherichia phage
vB_EcoS swi2
JOURNAL Unpublished
REFERENCE 2 (bases 1 to 47611)
AUTHORS Sui,B.
TITLE Direct Submission
JOURNAL Submitted (13-JUL-2020) College of Veterinary Medicine, Qingdao
Agricultural University, 106# Xiangyang Road, Chengyang District,
Qingdao, Shandong 266109, China
FEATURES Location/Qualifiers
source 1..47611
/organism="Escherichia phage vB_EcoS_swi2"
/mol_type="genomic DNA"
/db_xref="taxon:2769808"
CDS complement(120..2312)
/codon_start=1
/transl_table=11
/product="tail fibers protein"
/protein_id="QNR52424"
/translation="MATTPTNKPIPSEDPRDLKFNAGKIDEVVNSDAHYYTDRFGVR
R
WTIAGFQHTAEEAIRNYGYITMDSFEDGATLTLPNQTLRYEANGEYYRW
DGEFPKIVT
AGSTPETAGGIGVGSWVSVGDAALRTQLANTTGADLVGIMPYGTVQDA
IKWVVPEVFP
GGNASEKLQAAVNYAVANKTRLVASGVYDVTTPVTISGDIIIDASAGEFTF
NGIDYIF
HPLGAKSVEIIGGKFTANAYHTQRPQVIFNDYPDGLANLPTRVVLKDMQ
CFNCGVGYI
MVNCQDPTSVHISVDNNYCKTDDNTDQYISDAGMSGAQGEVYPYLMI
LGNTTASVDIG
TPRKSMFHVTNNTFDVFMQSGPNADLGKVGGTTIGGNVNGNLFCNRN
TECACEFDTFT
GGLEVSLTSNRFVNTAIKMMSMQFDSSTRVGLGGRSSISNNVFHFEENP

LNDFAIFLR
TSLVSISNNVFYYKGASIPSDQRIFNFIASQALNNSNNGFNGTWCAGISIT
GNTMQMV
LDPAVNTSLRLQCINPTDMSGAVISGNFMAGGVGMVLNARPERNRNV
WTGNFITSGLF
TAEDIWRMNSAFVGSGNYIGNGYDNTVNGIAMLSKTIPAVSDGSKIRITL
DHQILTGA
TSDRSLYLLHIKVTGGIKNNYATYIMSSGAHDATSRADATDLKPPIDQRL
NPGDTSIA
NLQSCFKAGYNGSGYIVLEALSTYSSANQPPTKLEYAIVPLSTNFPTI"
CDS complement(2352..4826)
/codon_start=1
/transl_table=11
/product="hypothetical protein"
/protein_id="QNR52425"
/translation="MPVIHVQKMPGTPKETGIVPAGTNLWKWLNKSNLPASIS
IAVNG
RVLGEDDELSFCLRDGDVVNVYCQPSGAIGDLIGAILKPVTKIFSFLTPKVS
TPKTDT
SSKTSPNTSLKAQTNIARNGEARPDNFGQIRAFPDLLQESLFEYINNIKYV
TEFMNFG
LGKYDVSSVRYSESNLGSLAGASYTIYQPGEVIPVVLEPYAFDDVDGQELY
GPNDTNS
VVIESATTTSVTSTDFAGGQIAVKIPKNSAFDYFVDLVMPHDVVFKLNITY
ALGGGTS
VTENVTLSGSLVSASETDDGNIPPVDYWYTFIINNINYSGAPISSLSGATIN
NTYFNL
TDNQPLVSGPYFSPIEGDQLWFHFVAQFGEDQGAIVKAEWWAVDDDN
VQIAGSYQSTT
YTFVAGGADTYYYTRKITPSYGSARYAVQFTRTNNSTQESIIQLDEVHSIV
TRRNVSY
PDDTVVKVIVRATENATGSRDRKYNALIMRHTIGYNRDTGTVRYTLAPSR
SFADAVLH
NWLITAGNPENTIDIVKLYEIADSLPDERLGYFDYTFDDEDKSIGERLQTIC
DAARVT
AFWDDGVMSFSRDEKREYPATVFNTRNTQSDGYKLSYDISLPGTYDGVNVE
YRDPTTN
KQANVYYRITNSGIVEGEPTKAKKFDMLYVRNRYQAVDRAILECRRLIYSR
RSMEIKA
LADGEWVNVGDMIQVVDMYDDVQQTGVIEARNGNALTTSEQLTADD
NLYVVITSSDGS
VSDRLPATVTGLHTFTCNLPSDFKLNIWDGTNVQSESRYVLSTEKELDTTL
WVVSQKN
PGSDGTTTLTMSEYSDDMYEYAIQSS"

CDS complement(5198..5668)
/codon_start=1
/transl_table=11
/product="hypothetical protein"
/protein_id="QNR52426"
/translation="MPSLRDYKAKRPNWALFDTITFYHSSFGYVRLVANVLDE
MVLGG
ETYLPVRMDITQSQQSNTPAINATVKFARLANDFKQYLKLWTGSGRIEPI
TALYQRFE
ETDTNTPLKPYRLYVSDVAMDGSDVTVTLSIKNPIKGNVAKLYDIAQFPG
LRNV"
CDS complement(5668..6141)
/codon_start=1
/transl_table=11
/product="hypothetical protein"
/protein_id="QNR52427"
/translation="MAIIPYPSWLPLAQRASKNLTFQTPFRQDVPAVGAPIFQKLTTD
ISSQWSLTWKFTLAEERAFIQWVRSPNYLNKANNWFTMMIDLGGSGLQ
EQTLHFTDYP
VQTSIDGGVVTWAGNVIAKKLNNTMDEFDDVLVELDYRWYSWLDEVV
NRDLPEYP"
CDS complement(6132..6245)
/codon_start=1
/transl_table=11
/product="hypothetical protein"
/protein_id="QNR52428"
/translation="MQQAITNRFSSGRTSCTYDCTISVLKYNNTRWRLSWP"
CDS 6278..7156
/codon_start=1
/transl_table=11
/product="hypothetical protein"
/protein_id="QNR52429"
/translation="MSKSTAEEFIIKARGIHGDKYLYDAVSYIKSSIKVKITCRKHG
L
FEQTPNSHLNGAGCPSCSGNKKKTTREFVADAIAKHGDSYDYSKVEYRG
GHKKVKIIC
KVHNFEFSQEANSHLNGCGCPVCAREKITASVTKGFNKFKKDAIIIHGNK
YHYDCLSY
INVTTKMRIKCPEHGWFTKTPDKHLQGQGCPHCSKGGFKLKEKAFVYFLF
SGNEIKVG
ITNNLRRRVCQLKKNTPFDFHVISKIKTIGSDALAIEKYYHKKYESAGLTGF
DGATEW
LKYSPELMDEIMNKAP"
CDS complement(7169..9547)

/codon_start=1
/transl_table=11
/product="tape measure"
/protein_id="QNR52430"
/translation="MAEEVGGIVYEVGMDVKGLKAGATTANKTLDDLESSLNK
TTGEF
NKLDKGARNAGSGMKNAGGAASGLKTSMSMLAGAISVSLIIEWGKRFLE
VADNMTQLQ
ARIARLSTDAKTANETFSTLANIASTTGASLSDTTKLWETLTSSLKEAGATNAQVLNL
TDTLQKIGRIGGSSTEEMANALRQFGQSIASGTIRAEEFNSILEQMPELAR
QIAAGLG
ISMGELRARMLDGKLTAEDALNAIQDRTSVVNAEFAKLPRSISQATGSLE
TSFAKMIA
SINEATGASSTFVSVIDSITSAINRLTGQTIGAAEVISDLTSTAEMFSRRARTW
SWIG
IDGWAAQNQAIAAVANQAATLVADMDAVTKSTGEAAKAQAHLTVPK
AAEKEKKKKATG
KSAEERQAESVAEKLEKLRQQTMLNATSTSELSREQAILNAQQSLGKSAT
QEQIKLAG
EYAAKIWDQKNALKEQAAAEKERQRVEQSYQGLRAIASPTAGIDSEYQQ
RMADLDAYA
AAYPQKITEIEQARAAIEAQYRQQRMDAMWAEWQQQSLGAQLFGTAL
DSAMSAASNSI
TGLLTGTMSVQDAMRSLGSTVLNSLVNSFVEMGVQWVKSAVMGQTA
QVAATATTTAAQ
TAGLATTTAASTAAAATTTAAWTPAAIVASIGSFGGAAAIGVGAVLGALA
MGIAGKRK
NGGPVSAGSMYEVGEGNAPEIFQASTGRQYMIPGNSGKVISNKDISGGG
GGVVVYNNV
YNNSSNASATSRATDNGDGSITIETFISDMNEGGPMSQSISRNFNTNRR
ATE"
CDS complement(9547..10284)
/codon_start=1
/transl_table=11
/product="hypothetical protein"
/protein_id="QNR52431"
/translation="MSNRTPLTEIGEMRISLSDRSFFFKPSFRAMNEIGTPKEIVE
VY
AKLNGIDYVAPLQHVEYLPFGAQMQVMKTISKPVYGRHVLSAAYIVMQS
CCEDDISVL
IGGWKPTPRGVRYVPGVMPIGGTGHLPGGTGGIIEIARSLMEHGIIGKSPL
KVPERLE
EQGKKTTNEFHASQYIISARTHFDMTRDEAENLSMTEFQMMIKNKYPEP
KGLTKEERA

AEYDQAKADRERMKALAERKAKKARNT"
CDS 10398..10712
/codon_start=1
/transl_table=11
/product="hypothetical protein"
/protein_id="QNR52432"
/translation="MINKKIIICGLSAVFLIGAMQVAVMLGFMKPLFMFSYSGAI
QSV
SLLFVIGLVIFVYLLPAFVALQRKHVNTTAICVLNIFAGWCFFGWVAALVW
ALVKSGD
KK"
CDS 10709..10852
/codon_start=1
/transl_table=11
/product="hypothetical protein"
/protein_id="QNR52433"
/translation="MNEQTKADLTFYTELYVDAGYDYEEAERMAKDLLRVIGVI
FDED
KVI"
CDS 10849..11049
/codon_start=1
/transl_table=11
/product="hypothetical protein"
/protein_id="QNR52434"
/translation="MSQWIKCSDRMPEEGEMVLTAFRGVVRTAVCKVIDNIGS
KIFVS
ELERCHGIPATHWANPPEPPQE"
CDS complement(11080..11796)
/codon_start=1
/transl_table=11
/product="neck whiskers protein"
/protein_id="QNR52435"
/translation="MAICANDKGVLVGRMTRLFLAEGCGDAVPEAEDWKYLG
STTSKG
VDYSPQTTTSEADTAGGFVSTLVTSSDMTISAEVEIRKNDPSDEFGFHRLV
EIYTTEL
KARRQPSLWVREVTGATIVTAYCNITSISYEGGTNDIVTGSLEFKVYDSDS
VTVESLE
PLKFTTDLQPTGTTGSPLTVVVEGGISPYTYVWRKDGVVVGGESGASLAS
PTAGVYTV
TVTDSSTDPEIIISTACTVS"
tRNA 12345..12418
/product="tRNA-Met"
CDS complement(12450..12857)

/codon_start=1
/transl_table=11
/product="hypothetical protein"
/protein_id="QNR52436"
/translation="MCIDIAILGFNMNMLELVDAYLQDAGLYDGWTSQLQF
WNDTGDG
SEQFIVLQSNGGTQVMDGLGGDFYFSLYVVGKQGQYNVADVDAKALEII
EYIKTHPID
SCVNYIQLQAPLGRPMLTEEKRPVHELLLRVVK"
CDS complement(12859..12954)
/codon_start=1
/transl_table=11
/product="hypothetical protein"
/protein_id="QNR52437"
/translation="MVATNHTDKGVKFIVACVSFRVPRLRDIGSV"
CDS 12995..13486
/codon_start=1
/transl_table=11
/product="HNH homing endonuclease"
/protein_id="QNR52438"
/translation="MISERDYIAIASRVKYDPITGLFTWAVSCGKISKGDIANYH
NDA
GYITLGKKRLRAHRVAWFIHYGYVPEHEIDHINNIRDDNRISNLREASDCE
NARNTKI
SKSNTSGYKGVHFCKYTGKWRATVKMHGKSYHLGRFSDKEAAHKAYCK
KVDELFLEFA
NHG"
CDS complement(13483..13881)
/codon_start=1
/transl_table=11
/product="hypothetical protein"
/protein_id="QNR52439"
/translation="MPAKLKGINEAIARTSQIVDEIIATKAVRALKSATYIIRTESA
T
LTPIDTSTLINSQFDTVEVSGTRITGKVGYSAKYALYVHNASGKLAGKPRS
NGNGTYW
SPNAEPRFLTKAADKTRSLVDSVIKKEMKI"
CDS complement(13883..14080)
/codon_start=1
/transl_table=11
/product="hypothetical protein"
/protein_id="QNR52440"
/translation="MKDEKLNLKNHKQSRNKSGSRIWFSAYVKPSGRYWKRK
ASKAAR

KSDDLSCGGAYKKCFGWMEWC"
CDS complement(14080..14421)
/codon_start=1
/transl_table=11
/product="hypothetical protein"
/protein_id="QNR52441"
/translation="MTSLANWSYTQPCTIWHKSGTDKYGKPTFDAPVSIMCD
YGFNDD
VSTDAKGNEIVQKNTFWTEYTGAKVGDYIIIGTVTEDDPLSAGANQILNVI
NYGNTFS
RAEPPDFALIT"
CDS complement(14418..14810)
/codon_start=1
/transl_table=11
/product="hypothetical protein"
/protein_id="QNR52442"
/translation="MTAPTPEELVSQMASRGMTITTTDASGILCLVASISECLEL
NYP
NDECRQNAIMLWASILISANTAGRYVTSQSAPSGASQSFAYGSKPWVAL
YNQMKLLDT
AGCTGDLVEDPDGSGKPWFAVVRGSKCK"
CDS complement(14791..15075)
/codon_start=1
/transl_table=11
/product="hypothetical protein"
/protein_id="QNR52443"
/translation="MNGALRQVAEQIISGTTGQVIDKAGYASIGTGIGLKVAEQ
TPVT
QSYFEAMIPHSLTEWAAVASILGALSLVIKNLFEMWWKVRESKRNDSTN
T"
CDS complement(15108..15548)
/codon_start=1
/transl_table=11
/product="hypothetical protein"
/protein_id="QNR52444"
/translation="MFGADVAIMIMYVLGFACTGMVAFLVFIPAMVMSVYLG
WVLVDS
FPAEYLYYLAQSMVWLFQAIALRKSTKMALCVLTMSLYEWLVAIESFVWE
FITPVETP
LHAQYAFIIIGTHLFILSITFKWGGEIGHYSWRGRHCFFADSNL"
CDS complement(15548..15667)
/codon_start=1
/transl_table=11
/product="hypothetical protein"

/protein_id="QNR52445"
/translation="MGSTNSPSRSRATGNTKTGGKTGAVKPNGSTSSPSRGKK
"
CDS 15785..16093
/codon_start=1
/transl_table=11
/product="hypothetical protein"
/protein_id="QNR52446"
/translation="MKDEFKGTKGQWTNHSLFTGEARTSIWSGDIYVAEVMS
VRESES
VKKANSNIIAAAPELLEALRQLRDYVEDVCAVSSDDCHEEHPLNLANKAIKKALGKK
Q
"
CDS 16090..16899
/codon_start=1
/transl_table=11
/product="hypothetical protein"
/protein_id="QNR52447"
/translation="MKTLSKIYSDKETRNGIAVNKTYLVPVEQIYLEPGYNIREA
DEQ
HVEYFAQCWESGQPLPALTVIPDEKGIRILDGQHRYLGALRAIERGAPIAR
IECKDFT
GDEADKIAFMVSSSQGKQLDPFERAKAYTRLKGFGWTNEEIAKKVGRSV
SDVQMHLSL
GDVPAEVKARISAGQISYANAVAVTREHGDDAVKVIDEAVEEAKAQGKD
KVTAKVLKS
KKIKPVDRLIELLKQADHVILPAGHFVAEDEEFIQIPVADIHEVMAILEKM"
CDS 16899..17102
/codon_start=1
/transl_table=11
/product="hypothetical protein"
/protein_id="QNR52448"
/translation="MNEHDDKLIAGWGDPLLAKKVHYFRDGKIISECGKWMF
GGERED
ISAWKHDAICKQCLKKYEKMEKM"
CDS 17102..17353
/codon_start=1
/transl_table=11
/product="hypothetical protein"
/protein_id="QNR52449"
/translation="MSKYEKLDSMIMDILDFKIPTPFMAIHFSDGIHSGIYAECE
KLA
TKPHEGFRVLDRRLQALRKKGLILSHGASKGWVKINHER"
CDS 17343..17516

/codon_start=1
/transl_table=11
/product="hypothetical protein"
/protein_id="QNR52450"
/translation="MNAEQFIEKQLRAKLPDIDQMAIDAAIQYYKRNQSAKKG
GIFEE
CLKVAKQHMIKVK"
CDS 17516..18052
/codon_start=1
/transl_table=11
/product="DNA polymerase III beta subunit"
/protein_id="QNR52451"
/translation="MKLKISKLLLESALIFQARNDVRYYLNGICFMPDGRIASTD
GHR
AFIGGNHDNNLTENVIIKIGKSPTKRYEHAIIDTKSKIATYHNEAGVMVGA
GICEEID
GRFPDIDRVIPKETKAADEIGFNAGYLVDIEKAAKLFNPKFCGVKFELKGNT
NAAVCC
LSAPSGETAKIVVMPMRL"
CDS complement(18086..18934)
/codon_start=1
/transl_table=11
/product="hypothetical protein"
/protein_id="QNR52452"
/translation="MAVRYDIFTTREDGEVILDELMSADWTALQVNVMPINSS
GAYTP
LVSGTVSVKVSPFESGNYWIDVNNNNYYGVALRLKIIKTELPAAVSSLRVS
VWRAGVS
PQSVVNLQPDVALSDITTQSYIERANKQGKLFTASRRVTDVAGGSNLDS
VFITGTKQV
IFNQRIIGYTGKGVVASIYRGAVAAGGTAAEINNPNDITPQAATAQLLTG
STVTNIGQ
LTVAATYSEGNASNQGQGNSQARLGEQIIMAPNTTYLLRITSLDTAVQNI
NAYVSWFE
DDTYLA"
CDS complement(18975..19172)
/codon_start=1
/transl_table=11
/product="hypothetical protein"
/protein_id="QNR52453"
/translation="MAKYEVIARGIFVKEKGKIRELQLGEVITEPDEHLLPKLRIMPE
LEKSFEVATPQEKTTKRKKAE"
CDS complement(19216..20286)
/codon_start=1

/transl_table=11
/product="hypothetical protein"
/protein_id="QNR52454"
/translation="MEKIIFTKDLVANSAVVADQWKHLTIDRKVFCNAEAELAK
TYGV
NATALVTKDYWRDVDNVTTRVFRNEAGQDMMADLMGIAANINIGKTV
AISRIASDAGK
VVRTLSGQEPEDLDKTRYDYTGDVIPIFKTGYSREWRELLGMQSEGFYPLL
DDQANVT
FNLRSDMAQYLLTGDQTLNVNGVYTGYGITNHPNTVQVNLNASGGLNI
DLQTATPDEI
VTFFNQDFQAILDAQNVFEQVTLWVSPAVRRSFMRPYSNAAGFKGGTV
EQYITQFGNG
RIGKIGTNFLLTGNHFVGYVRNDMYIRPRVAQPVSTYAAARVNPHDNFN
FLVWSAMGL
QVRKDFTGKSKVFNGYGTQTPV"
CDS complement(20289..20756)
/codon_start=1
/transl_table=11
/product="hypothetical protein"
/protein_id="QNR52455"
/translation="MATIRYGTIIGGPARKNDPQIREGIMNASLQPGALVTFND
DDKI
IAHATAGGQGFPYVLQHNYLGGGDVSEAVPANATGMAVQCEFGVTYH
ALVAQSSVLKK
GTPLASNGAGALKVAGDGDNILFYSYETYTVASDGAELVAVRRAGNAS
MPAGA"
CDS complement(20756..22168)
/note="gp7"
/codon_start=1
/transl_table=11
/product="hypothetical protein"
/protein_id="QNR52456"
/translation="MIVKIGDKWVVKSKDGSQQFGEYDTEEVAKKRLAEVEAF
KHMNN
KLQVNVLTTINSASNISEKIIDGHPHYVIKNVVPVVDDVVMNDGLYPGEEI
RKSYHGL
DGKPAPYNHPMIDGKYVSASMTRAANQFSVGAWIENSSHDGSKALVD
LYINIAVAERT
EHGQELIGRIEALKNSAEGAEPIHVSTGLLLNREAAEGTSKGKKYSWIARN
MEWDHLA
ILPPGVPGAGTPEDGVGIFATNGEQIERITVNLEDSTVPDESANKINYKSW
LHKVINY
ITNKSDLSFENISEQIRQILKAEVGDDVWPYIVAVYDDRVGFEIKGQIFQQ

FYIVEDD
VVKLVGERVKAVYKTELEPVKSTEGEISMTNEELQAVLAEALKPVQESLTAVN
QKLTD
IEAENVKLKEQLQANTEQEETAMRAAIIAELKLPESAVNALKGEALRETYA
LTSKPAA
LKGGFQPNHADDDFDMEAPE"
CDS complement(22363..22509)
/codon_start=1
/transl_table=11
/product="hypothetical protein"
/protein_id="QNR52457"
/translation="MIPLLWILSAYAFARVFEADTLYQMICYGALFCLSGAALAF
MDD
VISD"
CDS complement(22506..22694)
/codon_start=1
/transl_table=11
/product="hypothetical protein"
/protein_id="QNR52458"
/translation="MSEPKFPELPVEVQVALINAASTIAANQISAVGGRYNEKY
DFFG
IAYRKICDSLYKENRGRP"
CDS complement(22691..23017)
/codon_start=1
/transl_table=11
/product="hypothetical protein"
/protein_id="QNR52459"
/translation="MIIQLNDIMKADIIQLEDYDVQLAFEIETVDRQLQYADKK
NDRV
WHEKALKARDHMKRKRALIKTRLDKLYFGEERMIHGAILAQIRKEMPIGKF
MSYVHRA
KQEAGL"
CDS complement(23014..23394)
/codon_start=1
/transl_table=11
/product="hypothetical protein"
/protein_id="QNR52460"
/translation="MRWAINHKSGRTLFVTSDKFIANNRRKMGWIVEEMKMT
SRKQFE
GWAKENGMSLVYGDCDYVYSPTAWAWKAWQASRAEVEIESPDFIDSR
SALNKGYTVDY
SNGFGDAMDAYEMVIEQAGLEVKK"
CDS complement(23520..24008)
/codon_start=1

/transl_table=11
/product="hypothetical protein"
/protein_id="QNR52461"
/translation="MDYKSQIMRVIMNHPGATRAYIEKHCGGKHSSTTTHRLH
EMLAL
GFIRREKSVIRGGKWQYKYFISDDSAGIDGAIKCHLLDNSGAEVKEISVAT
GIDYRIV
KSRIRIMFHNGDVTRSYDHHKKLWRYSWREQEVNVSNLFNSLLRSARGH
HGKSQTQEA
RV"
CDS complement(23998..24249)
/codon_start=1
/transl_table=11
/product="hypothetical protein"
/protein_id="QNR52462"
/translation="MSAPHMPMTNDEMLLECPFCGDSHAYIDNDGPGAFYV
ACSQCGC
GTDEWPHQASAVKSWNTRGGHLYTADDFNQAAQERDYGL"
CDS complement(24246..24440)
/codon_start=1
/transl_table=11
/product="hypothetical protein"
/protein_id="QNR52463"
/translation="MAHDELYEESLIKRLNEVERTREWLECELREVRNRLQRKR
SQQK
DVIDWSGDAPKFNNLGEWLK"
CDS complement(24501..24698)
/codon_start=1
/transl_table=11
/product="hypothetical protein"
/protein_id="QNR52464"
/translation="MRYKEIAARYQKEVREVMEILNVREDTIKFVETAMCSLALE
AEV
AGREKADELISAVVYSSTSNG"
CDS 24798..24992
/codon_start=1
/transl_table=11
/product="hypothetical protein"
/protein_id="QNR52465"
/translation="MPRPRREPMDIITSIVEKRQPLTLRDVRYFARCYVALADM
PKDD
MYQMIRENFNVDENNRVTMK"
CDS 24989..25480
/codon_start=1

/transl_table=11
/product="hypothetical protein"
/protein_id="QNR52466"
/translation="MKKWKYLKGYEDDFVDHDTACLVVKSGSTGEIFYLSIDYA
GRIE
VIEGCGDVVIAYREPITDEQDLNDCIGAPEADATEQLITERGSRYGKFKDG
AAIMQEL
KFVMREVDGWHNLTPSQREALDMIQHKIGRILNGDPTYDDSWKDIAGY
ATLIVNELNG
EIK"
CDS 25480..25788
/codon_start=1
/transl_table=11
/product="hypothetical protein"
/protein_id="QNR52467"
/translation="MSFCDITIAQRNANFTNIADTSAQLVSLNSDGSAVLKIGT
ETAQ
FIVQNLSQANAKQVLISTGSVLFLAGNYNAPNLECSLVRIVETTAEESVDD
PTTLPAE
"
CDS complement(26024..26254)
/codon_start=1
/transl_table=11
/product="hypothetical protein"
/protein_id="QNR52468"
/translation="MFKSDLIVKLNKDSTWTLIEPPIYEFRHQEIKVPAGFRTDFASV
PRLPYLFAFIGDVGQKAAAVHDYLYATHSDRH"
CDS complement(26262..26744)
/codon_start=1
/transl_table=11
/product="inner-membrance spanin protein RZ"
/protein_id="QNR52470"
/translation="MMSILAKYWQPLAIIIIVAAGALWIRGEVVEYGDQRYAEG
KAQA
IAEQKAADEKEEQRRNAELQKIQANAQQRIDAARNDAVNAAAKSGRLQ
QQLANIRKQL
VGYSTAESIGNPAAETGVLLAQLLSESVERNRQLADYADRAREAGLACQ
KQYESLTRR
"
CDS complement(26300..26566)
/codon_start=1
/transl_table=11
/product="lysis accessory protein RZ1"
/protein_id="QNR52469"

/translation="MQNCKRFRLTHSKGLMLRAMMLLMLPLSLAGCSNNLQTSASNSS
DIPPLSPLAIQPQKPEFCLPSCSQNLSKEIDNWQITLTEQERRD"
CDS complement(26725..26964)
/codon_start=1
/transl_table=11
/product="hypothetical protein"
/protein_id="QNR52471"
/translation="MKKLSNWLLGAWISFCSLLQLWPDAMMHVWVMMPD
DLKAALPPI
VVKGVSYSIMLVGILGKMHGMRKENRRLRNDVNSR"
CDS complement(26961..27425)
/codon_start=1
/transl_table=11
/product="glycoside hydrolase"
/protein_id="QNR52472"
/translation="MDISKNMKAFLDMLAYSEGTDNGRQKTNNHGYDVIVG
GSLFTEY
SDHPRKLISLPKLGIKSTAAGRYQVLAKFYDAYKKQLRLPDFSPASQDAIA
MQLIREC
KATADVEAGRIADAIHKCRSRWASLPGAGYGQHEQKLDKLIQVYKEAGG
VVA"
CDS complement(27415..27693)
/codon_start=1
/transl_table=11
/product="muraminidase"
/protein_id="QNR52473"
/translation="MELSQRGMEALDVTDAVDISPYITAETTQNQFDALTSLA
TDIGI
DTFRKSTLLKKHNLRCFSCAVAHFILWGEKTGDKAKRKAEKEVYWYGY"
CDS 27799..28077
/codon_start=1
/transl_table=11
/product="hypothetical protein"
/protein_id="QNR52474"
/translation="MCDCMNKMEELLKERLMERVPSGSEVSSNVFDKTGWD
NQFISLS
SGKVFVMLKYRLAYRARKKNGELAKNLTRLESNVKMSYCPFCGEKQVD"
CDS 28080..28223
/codon_start=1
/transl_table=11
/product="hypothetical protein"
/protein_id="QNR52475"
/translation="MVMVKFKENGRCGVFGLKQIKIRPCGKVIAPFGLVQMRE
VEIVE

YIK"
CDS 28232..28525
/codon_start=1
/transl_table=11
/product="hypothetical protein"
/protein_id="QNR52476"
/translation="MEEFKGTHGPWNYCADEPDWVTDSNDNIAVARVTRYN
ADAEAQH
ANAKLIAASPDLLSALQQLLEIYDDNSGKVWTTSSKRRALDNAREAVNK
ALGE"
CDS 28618..28752
/codon_start=1
/transl_table=11
/product="hypothetical protein"
/protein_id="QNR52477"
/translation="MAKKLNEVHSWREGGQNSYEVFKLGDLYEVEGEYKEILS
DGETK
"
CDS 28749..29399
/codon_start=1
/transl_table=11
/product="hypothetical protein"
/protein_id="QNR52478"
/translation="MKLIDLLVKELPKRGGWPQNSLSITQDNDGSLCVWDTN
DPHYEG
FSWKHHTGNSLMHFWCEEAAMPLSSDHKESIVTYWQYKAALAASQKP
TWDGEGLPPVG
AKVEFFINPKFGYRNAWIPDAGTEMEVVAHKTTTDGNDVAVCYWDDG
GAGRSCCFIPE
SLKPLRTEAERRRDAFINAVLDGMRVIPCDLSLRDEVAVIYDAIAAGKIPG
VKLDD"
CDS complement(29483..30340)
/codon_start=1
/transl_table=11
/product="hypothetical protein"
/protein_id="QNR52479"
/translation="MDKVITRKYKQLRTRALELFRTIPNGQTNAESSGLYFYDFS
SAR
AATFMDELQALIDEILLEGDDLDHGRMWANVFIGDAYQAGTQKANSEL
SSLSPVYAEQ
RPISAILYSEPYLNRLQLAYTQGYSDWRGLSDYSRQQLASVIMEGIARGA
NPRDVEAD
IVKRVDVSHSYAKQIAQTEITGTLRQANRREVIEAREELGIETVMLWQSAL
MRTTRQT

HAARHGRFYTPEEIDTFYSENGNRYNCHCAQTPALLMDGKPVILESSQERLDKQREA
W
QSANKKPSK"
CDS complement(30414..31664)
/codon_start=1
/transl_table=11
/product="hypothetical protein"
/protein_id="QNR52480"
/translation="MFRYAYERHPAAAAGINRIINKCWQKYPEVVEDGEDDK
NSTPWE
LSINDMMKRAYPFIKEADKRNAINRYSAVILQIRDGRQWSEPVDITKTRRI
KDKSIVR
FIPVWEEQLRVSAWNNDETSEDYGMPEMYEYQESAVEDFDSDGKPDRS
VQIHPDRIII
LAEGSFDGSMFSGIPMLRAGYNSLIDMAKVSGSSAEGFLKNASRQLAVN
YTKDNVTPA
SLAQSMGVDIEELTDIMNENIEALNSGIDAAMFTMGADAKVLAVTPADP
KPTWEVAAN
QFAASMALPFTVIFGQQTGRLASDEDKMQEAMTAKQRRETWVDYVIS
MFVERMIQFGV
VDKAPANGYKVKWDDLLAPSELDKAELLSKLATANKSFFDAGQSALLTVDEARG
MVGM
EPIELDESYREDTPPEDENEDTPV"
CDS complement(31872..32120)
/codon_start=1
/transl_table=11
/product="hypothetical protein"
/protein_id="QNR52481"
/translation="MVTIIIGFVVMAFFAYMFIRNSAVADARIGFIKFFYEGDPD
GYA
AGKRFHEALPSYDEMLWKFWVWPLSKFYPAYRNRNRNK"
CDS complement(32131..32292)
/codon_start=1
/transl_table=11
/product="hypothetical protein"
/protein_id="QNR52482"
/translation="MALFWSFWIPLNFMFWWAMAAEVNDPRWIHFIVGSFF
AVVTGVV
PCLLYQYFK"
CDS complement(32292..32480)
/codon_start=1
/transl_table=11
/product="hypothetical protein"
/protein_id="QNR52483"

/translation="MSVYFIHAEVFVNNDCAAEKLSGVMVATNAVDALENFWRNDMVS
SLTSQGIKVVIDKFEKVE"
CDS complement(32480..32641)
/codon_start=1
/transl_table=11
/product="hypothetical protein"
/protein_id="QNR52484"
/translation="MIIEKTNECHSLYINTELVINLRQCDDWVQLDKKQAAQLI
EVLQ
SWVNGDEIE"
CDS complement(32650..33057)
/codon_start=1
/transl_table=11
/product="hypothetical protein"
/protein_id="QNR52485"
/translation="MFRNKLKKIIRDAADKKYTKKIDISFIDGAGLYNHACHLNA
VNK
ARDGGSCAVVEVVMINDDGATAHYINMQSDGSYVDYTLGWHWSGAD
YRFVRYVPFTEW
SDITGSLSRLKAELCKPVAKWQKLLMVTDGELC"
CDS complement(33084..33362)
/codon_start=1
/transl_table=11
/product="hypothetical protein"
/protein_id="QNR52486"
/translation="MSGELFKKGQIIPKRIGGLHVHRKARHRLVFGCDVLIDCG
NWIA
VPETGKSMVIAKKLGVRRNNGTYQSVEAAFKDVHFNANKIDYVVKICR"
CDS complement(33367..33573)
/codon_start=1
/transl_table=11
/product="hypothetical protein"
/protein_id="QNR52487"
/translation="MRSYAGFTQEEKEQVYSLARAGVPDDVICRRYDIDEDFLL
RVLD
DVFVNLQEKRGYKGICCKNDFLRG"
CDS 33734..33889
/codon_start=1
/transl_table=11
/product="hypothetical protein"
/protein_id="QNR52488"
/translation="MFETKKECEAYIAETYGADYVKFGIVTAQKVGPTVAKMLG
IKEG
YYPSNAY"

CDS 33879..34091
/codon_start=1
/transl_table=11
/product="hypothetical protein"
/protein_id="QNR52489"
/translation="MPTNYMPRCLREIPKAKVKPRNQAIKEAKIEAFNIAISIIKD
RC
RNEKSERIKSNMYAAVSDISRLRDEL"
CDS complement(34114..35538)
/codon_start=1
/transl_table=11
/product="terminase large subunit"
/protein_id="QNR52490"
/translation="MARKRLSALAIEKLEAQIDDEMTDVAESAIFGICDIQKNVI
KRL
RMTATGVDDVTNATTHADHLIPAKLERLLYPKRFKFIYGGRGSGKTRTIITILTE
RAR
FRPDRFACFREIQQSIEDSSYQELVDEIARKGESAEFRVINNEITHKKTKAKF
RFKGL
YRNQTTVKGFAGITVGWVEEAENVSQTSWDILVPTIRAANSELWCSFNP
NKETDPTWK
NWIAPYHSQMVDGIFENDEILIIECNYSDNPWFWDTPLPSAMEQMKRV
DFDRYMWIWE
GKFNKRSDEQVFGGKWRIDNFEVKPEWHGPYFGMDFGFSTDPTAMVE
VYIEELPGGRR
NIYINREYGKVGLEITDTPAAMEQSFPMAKRARWYADCARPETISHIKRS
GFDIHPCT
KWPGSVEDGVTWLRGCDSIIIHERCKEMQNEAVMYSYKVDKLTGNVLT
DIVDAYNHYW
DAVRYALNDHIVQRGSGMLIRRRR"
CDS complement(35528..35674)
/codon_start=1
/transl_table=11
/product="hypothetical protein"
/protein_id="QNR52491"
/translation="MDDEANNYKSKCDEVLLPRNSSARSVTFDKAMSDEEVA
TILQGL
ANGS"
CDS complement(35667..36086)
/codon_start=1
/transl_table=11
/product="hypothetical protein"
/protein_id="QNR52492"
/translation="MAKKLFSKENQPQNKRGKDKRKLLVEALERKGFSEEKLYD

TIVE
MAMIERDTSMMKELIVRFSPLPKPVAPVFEVDFPDDGTPVEKIDAVIRGIA
AGVIPVD
IGKTFAEVIRTGLDIAEVTELAARLERLEKLLEQQNG"
CDS complement(36235..36408)
/codon_start=1
/transl_table=11
/product="hypothetical protein"
/protein_id="QNR52493"
/translation="MQTTKQKVWQLAKQHELDDFIAKVAKTFPDALEIVHVQ
TQTENA
WCYAGKRDSDGVQ"
CDS complement(36395..36574)
/codon_start=1
/transl_table=11
/product="hypothetical protein"
/protein_id="QNR52494"
/translation="MFDDINAAMEFMWKRYWDCMMTCHYMMVQLGNRIE
VVPDNGVHN
IKCMCSTRDYANANH"
CDS complement(36567..36674)
/codon_start=1
/transl_table=11
/product="hypothetical protein"
/protein_id="QNR52495"
/translation="MTKHQRRRYTTGAKIFLAVYVLALVAAIAGVVHYV"
CDS complement(36721..37038)
/codon_start=1
/transl_table=11
/product="hypothetical protein"
/protein_id="QNR52496"
/translation="MTITKQRVEEIISRIKMYGHGAGYTAEEVYDLAVLALNLSN
IVK
LKRYDLNTGGCDTFSPNCCADMTEDHEGEFVMFDDVVKRFQFDIALSN
SKAWLKVNST
RSY"
CDS complement(37035..37451)
/codon_start=1
/transl_table=11
/product="Eae protein"
/protein_id="QNR52497"
/translation="MSEVKRYDISAFGSMSMDECGDGAYVNYEDYAELEAKC
AALAAE
NATLNDKMNRILVWPGIEFYSSAWEFCNLDGNDAIELMCDNKTPATDA

FLAEVRAQGV
EMAMEHMQSSGSLTFGDCYISLNEFAAQLRKGVDKK"
CDS complement(37448..37681)
/codon_start=1
/transl_table=11
/product="hypothetical protein"
/protein_id="QNR52498"
/translation="MAKVKTYEFWFVRNAMYACKTVKRARWWNKWLILSGC
IVLAKCK
FKAIDITDEDALTIAKIEFEEDGYYEEIIGVRV"
CDS complement(37718..37900)
/codon_start=1
/transl_table=11
/product="hypothetical protein"
/protein_id="QNR52499"
/translation="MAKTIYRREKLEQELGHVGAQNFMSKKARNAMESIRVN
RVVRVF
NGEGKRRVMDELIIVF"
CDS complement(37989..38084)
/codon_start=1
/transl_table=11
/product="hypothetical protein"
/protein_id="QNR52500"
/translation="MTGASFEIIASLVIVALIIIAVAVSKSGYKE"
CDS complement(38081..38227)
/codon_start=1
/transl_table=11
/product="hypothetical protein"
/protein_id="QNR52501"
/translation="MKIKDREEFEDAQAMARIAVERTNNSIPAEAFWNAAMQ
ALISAY
GLSK"
CDS 38770..39048
/codon_start=1
/transl_table=11
/product="hypothetical protein"
/protein_id="QNR52502"
/translation="MYPDGTKLMFAYELLSSMKSGELVGIHFENEYSSEIIKGEK
ENC
CCVVIDEKYRMTHNMFRSYCTRIANEQGMVIKTKTSESKKFMYIWRVL"
CDS 39048..40676
/codon_start=1
/transl_table=11
/product="hypothetical protein"

/protein_id="QNR52503"
/translation="MTDSKQIIELKESLKPTGKPDSGNPLDEIDVPEFREGYKIPP
GS
LGEFMCEIENHFENREEGEVYRLPGAIALAQVMAGRYIIGPSAKNKCATG
TFIVGRSG
AGKGAPSDFVKAYAENLGITPRVSKSTVTSLRQIKERLIEADGFLLYIADDC
PEHLQA
WSDTRSPLGETASWFRTSISGDWFPESPVVTLFQEKLASAQNPKLILSAA
QAQGWMIP
RIGESDGAIDYRRLAKMNHDIGRRLNHAMQCYDLCINEKGIQNVRFIPFI
TVTPEQGI
ATVRRWEKDGGMGRSLFIKGHEHMPELKNTPDMEINKTIINEWKPRIPG
GFFNVEYAN
DGVSKYYEMLRRRIDKSSNIPGVIGSVGPRSAQMVIELATLCAFADLSSRN
GMTPQIR
ECHIEWAYATVMNSMYDLRDYLEGEAEFDGLENTEWDNIVMKVKKCIES
KAFAEKPYI
SVVKNKLCRDRISKIISAADSNSIQVTPDKFTYEVIIAISENRHSPIELDPENP
SNIR
LSGGGSWSGLRMNSSVRNILSSAMKRMRFMRNLK"
CDS 40673..40864
/codon_start=1
/transl_table=11
/product="hypothetical protein"
/protein_id="QNR52504"
/translation="MRTISGELCRSEGLWHFRPYGYSTWFFWSGINKVWIRSN
YHVLN
DYWHRFCVGHVDISKMMIK"
CDS complement(40981..41934)
/codon_start=1
/transl_table=11
/product="DNA primase/helicase"
/protein_id="QNR52505"
/translation="MRYYWKDIEPKMLGNWQAAIMSIVNVDSRVFNGKHQP
CPSCSGK
DRYRFDDNFETKGDGGAICNQCGSGSGMNWLMKLSGMSFPEALEALGGFLNMHP
REKL
EAIRKELPKINYNDNFITEQEVAAIMAKTTRVAMNEWTLINGIGCDVNVT
RGKSGELI
AVEMMRADTMKPCNVAFIGMDGDLFRTFFRAGYNKDSAINGKLTRGAI
SPIGEDNGKF
IYLASDYADAWKCHYFTGAHVWCCWSPENMWEVVRSVSDETKARLRCIV
NYKFDELCA
AENAGLPVMLPDDADTIRMAKRIRRKIYDAGELIEKMSVIR"

CDS 42079..42654
/codon_start=1
/transl_table=11
/product="HNH homing endonuclease"
/protein_id="QNR52506"
/translation="MSLSELQKQNVAQESADCASTDRRKTQFITQKQIMEYLE
YNPET
GVFTAAKTHGTLWRKGKIVGHKNKAGYITITLLGKLRKAHRLAWIYVYGE
DIDGYEID
HINGDKSDNRICNLRISSHQQNMFNMKKKSTNKSGVKGVHFDKGCNKWR
AQTSINKKR
VHLGLFDTIESAEKAIREFMVANHKEFINLG"
CDS 42658..44580
/codon_start=1
/transl_table=11
/product="DNA helicase"
/protein_id="QNR52507"
/translation="MHKIDKMISEIDINLLKSCLDTGDIEPRPYQWLIYKLTGDVI
RH
YVGPSYVTASVGSGKSLMIAMIAKRFQEMGYSGMILSRQGEIVEQDAEEL
WSFGVRNS
LFSASLGRKSSTYPIICGSEGTVVNALFDKKDGSGNVIAKGVLSDFCPRFLLI
DENHM
VNDIDVVNNGDTQYAVIINELMKRCKDKHGHELRIIGYTGSPFRGTTSIKG
AFWKKEI
INIDTKYMVENGFLVPTIFGLHDVDSLHYDLSDFHGSDVDGTQDFTAEQL
KQMQKEIL
EQGTLTQKIMLKVMELTKNRNGVLITCAGKKHCQEAAKYLPEGSYSIVTE
DMGSKARR
KALKDAYTGRKKFTFQIAALTTGVNIPLWDTSCILRKIMSLTLLVQLLGRG
MRLLKKE
QIDAGYHKEDHLVLDFSGTMFELGQLYEDPILEEAEAQRSKRSGEQVPCP
KCGTMNSP
YARRCIGKDALSPDGRCEEFFSYIRCGFDKHGIRIFDDGCGTKNDPTARYC
RHCDHVL
RDPNAALNERAYTDNEWADVMDFKVQLTKDGEGILYRYWINRCDGKE
GWANEVFYPYG
GATHMKNMFKAKAVFPHLDDKSMAGKILKCQNAKQFMMYAGLIKAPK
RITHRINDKGR
DIIHRKEFKGEQSEAA"
CDS 44567..44983
/codon_start=1
/transl_table=11
/product="hypothetical protein"

/protein_id="QNR52508"
/translation="MKQLDSGIWVFDSGYRGECPKEEIDQMGYGTWMQHRF
PDVLWFH
VPNETGTSSRVQFVLKRQKMGVKTGIGDNVIMTPGVKHTCGMIEAKRRDK
SKSRVSKE
QSTVLTEMCRLGHYAAIAYGLDELKKATLFYFGLDE"
CDS 45033..45209
/codon_start=1
/transl_table=11
/product="hypothetical protein"
/protein_id="QNR52509"
/translation="MDIEIPDSFDAEWQCEMLRNLLVKLNELDDGGYVVSDG
YSLLDD
AMKIVEALREYSGD"
CDS 45210..46148
/codon_start=1
/transl_table=11
/product="exodeoxyribonuclease VIII"
/protein_id="QNR52510"
/translation="MKVYFNNELTNEQYHADTEHINGSGLWNIYDRCPAAW
RYKDEED
EQSKALIFGTGSHTALLEPERFDAEYARMPTKEDFGDDLLVTVSDMNSW
AKERGIKGL
SGKSKAEVIKIIRATGEPVKIYDEERLIAEINANGRTLLEGDDYDVIQQMRA
VIHANS
YYSSLLAGSYAEVSILGELYGEKAKVRFDCLTKGGDIIDYKTAVSAKPDEFF
RHAARL
GYFMKMAMQHDMFVAAYGHAPRSVNLLVQEKKAPFIPALILLTEEQLRI
GRIQLNGAM
EIYKACKKANSWPGYSMGNPVIEMETPEWFKKQFNL"
CDS 46166..47206
/codon_start=1
/transl_table=11
/product="recombinase"
/protein_id="QNR52511"
/translation="MGILNIKPAERSGSRVVIGISGQSGSGKTYSALKLARGMV
DSPE
EIGFLDTENGRGRLYSNILDGKFLHADMYAPFSPARYRQAIEEFQVAGVK
VLVIDSGS
HEWEGEGSCTEIAEKPLLNGKKMADWKRAKAEHKKFMNAMLQSNMHII
VCLRARQKTD
FANPKEPVSLGLQPVCEKDFMFEMTVSMMMHDGGKIQEFTKLPEELRPI
FFESGRESV
RHGYIGEAHGRGLIKWVDSGVKVDEEFESWRSRLQLSAAKGMEGLKEEA

KSIPDNLKD

KIRAIWPSLAASAAEYDRIESFINDEQLSPVVITPQDNFNPAKLAKQQPQ

QPEQQEET

KTEHKPTPIEGF"

ORIGIN

1 cgacaacaac agccaccaaa acaacaaagc acacaaggag gtaatgagac tccgatggac
61 tttgatgatt caataccatt ttgatgataa aactaacccc gcataagcgg ggttttatat
121 taaattgttg ggaagtttgt tgataatggt actattgcat attcaagttt tgttggtggt
181 tgatttgccg agctgtatgt tgaaagagcc tcaagcacaa tatacccact cccgttgtag
241 cctgctttga agcaactttg caggtttgct atggatgtgt ctccggggtt tagacgctgg
301 tcaataggtg gtttaaggtc agttgcgtcg gctcttgatg ttgcatcatg agcaccagaa
361 gacatgatat atgtagcgta gttgtttttt atcccaccgg ttacttttat gtggagaagg
421 taaagtgaac ggtcagatgt ggcccctgtt aatatctgat ggtcaagagt aattcttatc
481 tttgaaccat ctgatacagc tggtatagtc ttgctcaaca ttgctatgcc attaacagta
541 ttatcataac cattgccgat gtagttccct gaacctacaa aagctgagtt cattcgccag
601 atatcttctg cagtaaataa ccctgacgta atgaagttac ctgtccatac gtttctgttt
661 ctttctggtc tggcgttaag caccattcct actccgccag ccataaaatt acctgatata
721 actgccccag acatatcggt cgggttaata cattgcaacc ttagagacgt gtttacggca
781 gggtctaaca ccatttgcat tgtgttaccg gtgatactaa tacctgcgca ccatgttccg
841 ttgaaaccat tgttactatt gttgagcgcc tgtgatgcta taaagttaaa tatcctctga
901 tctgatggaa tgctcgcacc tttgtaataa aaaacattat tagatatgct aacaagtgat
961 gtccgtaaga atattgcaaa gtcatttaac ggattttctt caaaatgaaa tacgttattc
1021 gatattgaag atctaccacc taatccgacg cgagtgctgc tatcaaattg cattgacatc
1081 attttaatgg cagtgttaac aaaacggttt gatgttaaag atacttcaag accaccagta
1141 aaagtgtcaa attcgcatgc acattccgta ttcctgttgc agaaaagatt gccgttaaca
1201 ttgccgccta tcgtagtacc accaacttta cccaaatctg cattaggacc actctgcata
1261 aaaacatcaa aggtattatt ggtcacatgg aacattgact tgcgaggcgt cccaatgtcg
1321 acggatgcag tcgtgtttcc aagaatcatc agatatggat atacctcacc ctgtgcacct
1381 gacattcctg catcagaaat atactggtct gtgttgtcat cagtcttgca gtagttatta
1441 tcaactgaga tatgtacgct agttgggtcc tgacagttaa ccatgatgta gccaacaccg
1501 cagttaaagc attgcatatc ttttaacacc acgcgcgttg gcaaattcgc cagcccatca
1561 gggtaatcat tgaaaattac ctgaggacgc tgggtgtggt atgcatttgc ggtgaactta
1621 ccgccgataa tctctactga ttttgcgcca agcggatgga aaatataatc aatgccattg
1681 aaggtaaatt ctccagcgga agcatcaata atgatgtctc ctgagatggt gacgggggtt
1741 gttacgtcat acacgcctga tgcgacaagt cttgttttgt ttgccaccgc ataatttaca
1801 gctgcctgta atttttctga cgcattacct ccggggaata cctctgggac aacccatttg
1861 atggcgtcct gtaccgtgcc atatggcata atcccaacaa gatctgcgcc agtcgtgttt
1921 gctaactgtg ttctcaaagc tgcatcacca acactaaccc aggaaccaac accaatacca
1981 ccagctgttt caggtgttga accagcggtg acgatttttg gaaactcccc atcccagcga
2041 taatattctc cgttcgcttc ataacgcagg gtctgattgg gcagcgtcag cgtcgcacca
2101 tcttcgaagc tgtccatcgt aatgtagcca tagttgcgga tggcctcttc tgcggtgtgt
2161 tgaaagccag caattgtcca gcggcgtaca ccgaatctgt cggtatagta atgcgcatca
2221 gagttgacaa cctcgtcgat ttttccggca ttgaacttaa ggtcgcgtgg gtcttctgat
2281 ggaattggtt tattagttgg tgtggtggcc attataataa atgctccttt gtgaatttga

2341 tgtatattgt atcacgatga ctgaatggca tattcgtaca tgtcgtcgct gtattcgctc
2401 atggtcagag ttgttgtgcc gtcacttccc ggattcttct ggctgacaac ccacaacgtg
2461 gtatcaagct ctttctcagt gctcagcacg taacgagatt ctgattgcac gtttgtacca
2521 tcccagatgt ttaacttgaa atctgatggc agattgcagg tgaatgtatg caatccagtt
2581 actgtggctg gcaatctgtc tgatacgctg ccgtcagaac tggtaatcac aacataaaga
2641 ttatcatcag ccgttagctg ctcgctggtt gtgagtgcgt tcccgttacg cgcttcaata
2701 acgccagtct gttgcacgtc atcgtacata tctacaacct gaatcatgtc acctacgtta
2761 acccattcac catctgccag cgctttaatt tccatgctgc gacgagagta aatcagcctg
2821 cgacattcaa ggattgcccg gtcaacagcc tgatatcgat tgcgaacata aagcatgtcg
2881 aatttcttcg ctttagttgg ctcgccttct acaattccgc tgttggtaat tctatagtaa
2941 acgttggcct gcttattcgt tgttgggtct cgatattcga cgttaacgcc atcataagtg
3001 ccgggaaggc tgatatcgta actcagtttg tagccatcgc tctgcgtgtt tctggtattg
3061 aatacagtag ctggatattc gcgtttttca tcacgcgaga aactcatcac tccatcatcc
3121 cagaatgcgg tgacacgcgc cgcatcgcag atggtctgca ggcgttcacc gatgctctta
3181 tcctcatcgt caaacgtata atcgaaatac ccaagtcgct catcaggcag gctatcggca
3241 atttcataca gcttcacgat gtcgatcgtg ttttctggat tgccagcggt aatcagccag
3301 ttatgcagaa cagcatcagc aaagctacga gatggtgcaa gtgtatagcg caccgtgcca
3361 gtatcccggt tgtatccgat ggtatgacgc atgattagcg cattatattt cctgtcacgg
3421 cttcctgttg cgttctccgt tgcgcgcacg ataaccttaa ccaccgtatc atctggatac
3481 gaaacgtttc tgcgcgtgac gattgaatgc acttcatcaa gctgaatgat tgattcttgg
3541 gtgctgttgt ttgtccttgt aaactgaact gcgtatctgg cgctaccata tgatggcgtt
3601 atttttcttg tgtaatagta ggtatctgct cctccagcca caaaggtata tgtggttgat
3661 tggtatgaac ctgcaatctg cacgttatca tcatccaccg cccaccactc agctttgaca
3721 atagcaccct gatcttcacc aaattgagca acaaagtgga accacagctg atcaccttct
3781 attggtgaga aatacggacc tgaaacaaga ggttggttat cagtaaggtt gaagtaagtg
3841 ttattgatgg ttgcgccact caaggatgat atcggtgcgc ctgagtagtt gatgttatta
3901 attataaatg tataccagta atcaacgggt ggaatatttc catcatccgt ttctgatgct
3961 gacacaagac tacctgacaa ggtgacgttt tcagtgaccg acgtgccgcc gccaagcgcg
4021 taagtgatgt ttagtttgaa aaccacatca tgaggcataa ccaaatcaac aaagtaatcg
4081 aaagcggaat tttttggtat ttttacagct atttgtccgc cagcaaagtc agtgctggtc
4141 accgatgttg ttgtggctga ctcaatgaca acagagttag tgtcgtttgg cccatacagt
4201 tcctgaccgt caacgtcatc aaatgcgtaa ggttcaagaa caacagggat aacttctcca
4261 ggctgataaa tggtgtaact agcgccagcc agagagccga ggtttgattc cgaataacgc
4321 acagaagata catcatactt accgaggcca aagttcatga actcggtgac gtatttaata
4381 ttattgatgt attcgaataa tgattcctga agtaagtcag gaaacgcacg aatctgtccg
4441 aagttatcag ggcgcgcctc gccgttacga gcaatgttag tctgtgcctt caggctggta
4501 tttggtgatg tttttgagct ggtatccgtt ttgggtgtgg ataccttcgg cgtaaggaag
4561 gagaaaatct tcgttactgg tttcagtatc gcaccgatca ggtcgccgat tgcgccggat
4621 ggctgacaat aaacgttgac aacgtcgcca tcgcgcaggc agaaggaaag ctcatcatct
4681 tcaccaagca ctctgccatt taccgcaatt gaaatgctgg caggaagatt tgatttattc
4741 agccacttcc acaggtttgt gccagctggc acaattcccg tttctttcgg cgtgcccggc
4801 atcttctgaa catgaattac tggcataggt gagaaacctt aactttgttg atattttttc
4861 gagtgttcgc agcctgtcaa atctgactgc cgttttctct cgcgcatgaa gtattctatc
4921 acgcccccat atcatggcga tgtgcacagg gacgctgccg cgatatgcca caacaacatc

4981 gcctaccgat ggcgactgcg tatccctcca gaacgtcacc tcgttatcga agcaggtaac
5041 aaaagagccg ccattatcgt agctgtcgtc atgatgaata ttgatgccac ggcacagtcg
5101 gtaataaagc accaccagac cccagcaatc aacggcgtca acatggcagc acctgtcctt
5161 gtatggctta ccaaacatta actgcgcaaa ttcttcatca gacattacgc agcccgggga
5221 attgagcgat gtcataaagt tttgccacgt ttcctttgat tgggtttttg attgacagcg
5281 taacggtaac atcagaacca tccattgcca catcgctgac atacaggcga tatggcttta
5341 atggtgtgtt ggtgtcagtc tcttcaaatc gctgatacag cgcggtaatt ggctcaatac
5401 gaccggaacc agtccacaac ttcaggtatt gcttgaagtc attagccaga cgtgcaaact
5461 tgacggttgc gttaatggca ggtgtattcg actgctggga ctgcgtgatg tccatgcgca
5521 ctggcaaata ggtttcgccg ccaagcacca tttcatccag aacgttagcc acaagacgca
5581 cataaccaaa tgacgagtga taaaacgtta tcgtgtcgaa cagcgcccag ttagggcgct
5641 ttgctttgta atcgcgtaat gatggcatta tgggtactcc ggcaggtcac ggttaactac
5701 ttcatccagc cagctatacc atctgtaatc cagttcaacc agaacatcat caaactcatc
5761 catcgtgtta ttgagtttct tagcgataac attgccagcc cacgttacca cgccaccatc
5821 aatgctggtt tgcaccggat aatcggtaaa gtgcagcgtc tgctcctgca atccgctgcc
5881 gccgaggtca atcatcatgg tgaaccagtt gtttgccttg ttgaggtagt ttgggcttcg
5941 aacccactgg ataaatgcgc gttcttctgc cagtgtaaac ttccacgtca gtgaccactg
6001 cgaacttata tcagtcgtta atttttgaaa aataggcgcg ccaacagcag gaacatcctg
6061 tctgaatggg gtttgaaatg tcaaattctt gctggccctc tgagctaaag gcaaccagct
6121 tggatagggt attatggcca tgatagtctc catcttgtgt tgttgtattt tagcacgctg
6181 atggtacaat cataagtgca gctagtccgg ccagatgaaa agcgattggt tatcgcctgc
6241 tgcatcactt caaaccacct ttaaccgagg attcaatatg agtaagtcaa ccgctgaaga
6301 atttattata aaagccagag gtattcatgg tgataaatat ctttatgatg ctgtttctta
6361 tataaaaagt tcgataaagg taaagataac atgccgtaag catgggctat ttgagcagac
6421 tccaaactct catttgaatg gagctggatg cccttcttgc tctggaaata agaagaaaac
6481 aacgcgtgaa tttgtggctg atgcgattgc aaagcatggt gactcttatg attattctaa
6541 agttgaatac agaggcggtc acaagaaagt aaaaataata tgcaaagtac ataattttga
6601 gttttctcag gaggccaaca gccaccttaa tgggtgtggt tgccctgttt gcgcaaggga
6661 gaaaattaca gcatctgtaa ctaaggggtt caataagttc aagaaagatg ccattataat
6721 tcatgggaat aagtaccatt atgattgctt gtcatacatt aatgtgacaa caaaaatgag
6781 aataaaatgc ccagagcatg ggtggtttac aaagacgcca gataagcatc tccaagggca
6841 aggttgccct cattgctcca agggaggttt caagctgaaa gaaaaggcct ttgtttactt
6901 cttgttctca ggaaacgaaa ttaaagttgg gatcactaac aatcttaggc gtagagtttg
6961 tcagttaaag aaaaacacac catttgattt tcacgtcata tcaaaaataa agacaatagg
7021 tagtgatgcc cttgctattg aaaagtacta tcataagaaa tacgaaagtg caggactgac
7081 agggtttgat ggcgcaacgg agtggcttaa atattcacct gaattaatgg atgagataat
7141 gaacaaagcc ccgtaagggg cttatttttc actctgttgc tctcctgttg gtattaaagt
7201 ttcttgagat agattggctc atcggtccgc cttcattcat atcggatata aatgtctcaa
7261 tagttatcga gccatcaccg ttatcagtgg ctctgcttgt tgcgctagca ttgcttgaat
7321 tattgtaaac attattatag accacaactc cgccacctcc accggaaata tccttattgc
7381 tgataacctt tcctgagttg ccaggtatca tgtattgcct gcctgtactc gcctggaata
7441 tttctggtgc atttccctcg cccacttcgt acatacttcc tgcactaaca gggccaccat
7501 tttttcgctt gccagcaatt cccatcgcca gcgccccgag aacagcacct accccaattg
7561 cagccgctcc accgaaagaa ccgattgatg cgacgattgc cgcaggagtc catgctgccg

7621 ttgtggtggc tgccgctgca gttgatgctg ctgttgtggt tgccagtcct gccgtttgcg
7681 ccgctgtggt ggtagctgtt gccgcaacct gcgcagtctg ccccataacg gcggatttaa
7741 cccactgaac gcccatttca acaaagctgt ttaccagaga attcagcacc gtagagccga
7801 ggctgcgcat tgcatcctga acgctcattg tgcctgtgag caatccagtg atgctgtttg
7861 atgctgcact catcgctgaa tcaagcgccg tgccgaacaa ttgcgccccg aggctttgct
7921 gctgccattc agcccacatc gcatccattc tctgctggcg atactgcgct tcaattgctg
7981 cgcgagcctg ctcaatctcc gtgattttct gcggatatgc tgcggcgtag gcgtcaaggt
8041 cagccatgcg ttgctggtat tcgctatcaa tgccagccgt tggcgacgca atggcgcgta
8101 atccctgata tgattgttct actcgctgtc tttccttctc agctgctgcc tgctccttca
8161 gtgcattctt ctggtcccaa atcttggcag cgtactcgcc agccagtttt atctgctcct
8221 gagttgccga cttgccaagc gattgctgcg cgttgaggat ggcttgctcg cgggatagtt
8281 cgctggtaga tgtggcattg agcatggttt gctggcgtaa tttctccagc ttttcagcca
8341 ctgactcagc ttgccgttct tctgcacttt ttcctgttgc ttttttcttt ttctctttct
8401 ctgctgcttt tggcactgtc aagtgcgcct gcgcctttgc cgcttcccct gtcgatttgg
8461 tgacagcatc catatccgcg accaatgtgg ccgcctgatt tgctaccgcc gctatggctt
8521 gattctgcgc agcccaacca tcaattccaa tccatgacca tgttcttgct ctacgactga
8581 acatttctgc tgttgatgtc aaatcagaaa taacctctgc cgcacctatg gtctgaccag
8641 ttagtctgtt gattgctgag gtaatagagt caataactga aacaaaggta gaactggctc
8701 cggttgcttc gtttatgctt gctatcattt ttgcaaatga tgtttcaaga ctgccagtcg
8761 cctgagatat agagcgcggt agctttgcga actcagcatt gacgacactc gtcctgtcct
8821 gaatggcatt cagtgcatct tctgctgtca gtttgccatc gagcattctt gcgcgaagtt
8881 cacccattga aatgccaaga cccgccgcaa tctggcgagc aagctcaggc atctgttcaa
8941 ggatggagtt aaattcttcg gcgcggattg tgccagatgc gattgactga ccgaactgac
9001 gtaatgcatt cgccatttct tcagttgatg acccacctat tcgacctatt ttttgcaggg
9061 tatctgtaag gttcagaacc tgcgcatttg tcgctccagc ctctttcagt gatgacgtca
9121 atgtttccca caacttcgta gtgtcactga ggcttgcgcc agtggtcgag gcgatatttg
9181 ccagtgtgct gaatgtttcg ttcgcggttt tcgcatcagt tgacaggcga gcaatccttg
9241 cctgaagctg agtcatgtta tcggcaacct caaggaatcg ctttccccat tcgataatca
9301 gtgacacgga tattgcgcca gccagcatcg acatgctggt tttaagccct gaagctgcgc
9361 cgccagcatt cttcattccg ctgccagcat tgcgcgcgcc tttatccagc ttgttgaact
9421 ctcctgtagt cttgtttagt gatgattcaa ggtcatctaa agtcttattg gctgttgtcg
9481 cgccagcttt caggccttta acatccatcc cgacttcata gacaattccg ccgacttctt
9541 cagccattat gtgttcctcg ctttttcgc tttgcgttca gccagtgcct tcatgcgctc
9601 acggtctgct ttagcctgat cgtactctgc cgcgcgttct tctttcgtta accctttcgg
9661 ctctggatat ttattcttaa tcatcatctg aaactctgtc atggacaggt tttcagcctc
9721 atcgcgcgtc atgtcgaaat gcgtgcgtgc tgagatgatg tattgcgatg catgaaattc
9781 gtttgtggtt ttcttgcctt gctcttccag acgctcagga actttgagtg gtgacttgcc
9841 gatgatgccg tgctccatta atgagcgggc aatctcaata attccacctg ttccccctgg
9901 caaatgccca gtgccgccaa ttggcattac tcccggcaca tatctcacac cacgcggagt
9961 tggcttccat ccaccaatca gcacggaaat atcatcttca cagcatgact gcatgacgat
10021 gtaggcggcg ctcagtacat ggcgaccata cacgggcttg ctgatggtct tcatgacctg
10081 catctgcgcg ccaaatggca ggtattcgac gtgctgcaat ggcgcaacat aatcaatgcc
10141 attgagctta gcgtacacct cgacgatttc ctttggtgtg ccgatttcat tcatggcgcg
10201 gaatgatggc ttaaagaaaa aactcctgtc agaaagcgag atgcgcatct ccccgatttc

10261 tgttagtggc gtgcgattgc tcatgttttg catcctgaat ttgactgatg ttgattatat
10321 catctcagtg gtgttgacac ctgctaggtg gtgatgtaga ttcaagtcat ggaagcagat
10381 gaggaatgag gtgatttatg attaataaga aaattattat ttgtggtttg tcagcggtat
10441 ttctgattgg cgcaatgcag gtagctgtca tgcttgggtt tatgaagcct ctattcatgt
10501 tcagttatag cggagcaata cagagcgtat cactgttatt tgttattggt ttagttattt
10561 ttgtgtacct tttgcctgca tttgtggcgc tacaacgcaa gcatgtaaac acaacagcaa
10621 tttgcgtgct gaatattttt gctggctggt gtttctttgg ttgggtggct gcactggttt
10681 gggctttggt taaaagtggc gacaagaaat gaacgaacaa acaaaagctg acctgacttt
10741 ctacactgaa ttgtatgttg atgctggtta tgactacgaa gaagcagagc gaatggcaaa
10801 agacctgctt cgtgtgattg gtgtgatttt tgatgaggat aaggtgatat gagccagtgg
10861 attaagtgta gtgataggat gcctgaagaa ggcgagatgg ttctcacagc atttcgtggt
10921 gtcgttagaa ctgcggtatg taaagttatc gacaacattg gcagtaagat ttttgttagt
10981 gaattagaac gctgtcacgg cattccagct acccattggg ccaatccacc agaaccacca
11041 caagaataaa acaaagcccc tttcggggct ttttctttat caggatacag tgcaagctgt
11101 actgatgata atttcagggt cagtagatga atcagtcact gtaacggtat atacgccagc
11161 cgtaggactt gccagcgatg cgcctgattc accaccaaca actacgccat ctttgcgcca
11221 cacgtaagtg taaggagaga tgccaccttc aacaacaacc gttaacgggc ttccagtcgt
11281 gccagttggt tgcaggtcag tagtgaactt cagaggctca aggctttcga cggtaacgct
11341 gtcagaatca tacaccttaa actcaaggct tccggtaacg atgtcgttag tgccgccttc
11401 gtagctgatg ctggtgatgt tgcagtacgc ggtaacgata gtcgcaccag tcacctcgcg
11461 aacccacaag gaaggctggc gacgagcttt cagttcagtg gtgtaaatct cgaccagacg
11521 atggaagcca aactcatcgc tcgggtcatt cttgcggatt tccacttctg cgctgatggt
11581 catatcagag ctggtaacga gagtggaaac aaagccgcca gcggtatcag cttcagacgt
11641 ggtagtctgc ggtgagtagt caacaccctt actggtggtt gagcctaaat acttccagtc
11701 ttctgcctcc ggaactgcgt caccgcatcc ttcagcaagg aacagtcgag tcatacgacc
11761 gaccagaacg cccttatcat ttgcacaaat agccatttcg atctccgaat tgtgttagct
11821 gctaacgtgg tgattatatc acaggtgttg acagtgatta ttttgtggtg tagattgtat
11881 ttcagatagt tttcgtgagc gactttgcgg actttttaga aactgatcac aaagataaat
11941 gcaaacgaag aaatgtatct ggccgtagct taacagctaa acacaagtga ggtattccag
12001 ttcctcatca acgaatcttg cgcactggcc cggtgtgatt aataatgggc acacaacgga
12061 aagagcatta ctggtgacgg agactagcga ttagctatat gcgaaagttc tagaagccaa
12121 ggcggttcga ctccgcaagt gctctttccg gtgtggtgaa tgcgcaggct gatgcgcgca
12181 ggagagcttc ggaagaacaa ggtgcctgta tacaagccgg agatcagcac cggccaccac
12241 aatacttaac atccagcatt atcgcaatca tatgtagggg tatgtatggg ttacggggct
12301 ggatgttaag aaagcacgct ggcaatgctt aaaccagcac ttatggacgc gtagcttaat
12361 tggttaaagc aaccgactca taatcggctg attgaaggtt caaatccgtc cgtgtccacc
12421 aaattaaaag ccgctttatg cggctttatt tatttcacga cccgtaaaag caactcatgc
12481 acaggacgtt tttcttccgt cagcattggc ctaccaagtg gcgcttgcaa ctggatgtag
12541 ttaacgcatg aatcaatcgg atgcgtcttg atgtattcga taatctccag cgccttcgca
12601 tcaacatcag caacgttgta ctggccttgc ttgccaacaa catacagcga gaaatagaag
12661 tcaccgccga gtccatccat cacctgcgtg ccaccattgg attgcagaac aataaattgc
12721 tcacttccat ctccggtatc attccagaac tgcaattgcg aagtccagcc atcatacaat
12781 ccggcatcct gaagatatgc atcaaccagc tcaagcatgt tcatattaaa ccccaatatt
12841 gcaatatcga tgcacatatc atacactacc tatgtcacgt aggcgtggaa cccgaaacga

12901 cacacaggca acaatgaatt taacgccctt gtcagtatgg tttgttgcaa ccatgttcca
12961 cctgacaggg gcgtttccct tttggagtga gtaaatgatt tcagagcggg attacatagc
13021 aatcgcgtca agggttaaat acgacccgat taccggattg ttcacctggg ctgtaagttg
13081 cgggaaaata tcaaaaggtg atattgcaaa ttatcacaac gacgcaggat acataacact
13141 tgggaagaaa agattaaggg cgcaccgagt ggcttggttt atccattatg gatatgtccc
13201 tgagcatgaa attgatcaca taaacaatat tcgtgatgac aatagaattt caaacctgag
13261 ggaggccagt gactgcgaga acgcaaggaa cacaaagata tctaaatcaa acacgtctgg
13321 atataaagga gttcattttt gcaagtacac gggaaaatgg agagccacgg taaagatgca
13381 tgggaaaagc tatcatcttg gtaggttttc agacaaagaa gccgcccata aagcctattg
13441 caaaaaagta gatgaactgt ttttggagtt cgcaaaccac ggctaaatct tcatttcttt
13501 ttttatcact gaatcaacga ggctgcgagt tttatcggca gcctttgtta agaatctagg
13561 ttcagcatta ggcgaccagt acgtgccatt accattactg cgtggtttac ctgcaagttt
13621 accgctggca ttgtggacgt acagcgcata tttcgcagaa tagccaacct tgccagtgat
13681 tcgcgttccg ctaacttcca cagtatcaaa ctgactgtta atcagcgttg atgtgtcaat
13741 tggcgtcaac gtggctgatt cggtgcggat gatatatgtc gctgacttca gagcacgcac
13801 cgcttttgtg gcgattatct cgtccacaat ctgcgatgtt ctggcgatgg cttcgttaat
13861 acctttgagt ttggctggca tgttagcacc actccatcca tccaaagcat tttttgtatg
13921 ccccaccaca ggataaatca tcactttttc ttgcggcctt ggatgccttt cttttccagt
13981 acctgccaga cggttttaca tacgcactaa accagattct tgaacctgat ttgtttctgc
14041 tctgtttgtg gttcttgaga tttaattttt catctttcat tatgtaatca acgcaaagtc
14101 aggtggctca gcgcgactga acgtattgcc atagttaatc acattcagaa tctggtttgc
14161 gccagccgac agcgggtcat cttctgtcac cgtgccaatt atgatgtaat cacctacctt
14221 agcaccagtg tattctgtcc agaaagtatt cttctgcaca atctcattac ctttcgcgtc
14281 agtcgataca tcatcgttaa agccataatc gcacatgatg cttactggcg catcaaaagt
14341 tggcttgccg tacttgtcag tgccgctttt atgccagatg gtgcatggtt gcgtatagct
14401 ccagttagcc agcgaagtca tttgcattta ctcccacgca caaccgcaaa ccacggttta
14461 ccgcttccat cagggtcttc caccaaatcg ccagtgcatc cggctgtatc cagtagcttc
14521 atctgattgt acagcgccac ccacggctta ctgccatacg cgaatgattg tgatgcgcca
14581 gacggtgcgc tctgactggt aacgtagcga ccagcggtat ttgcgctaat cagaatggaa
14641 gcccacagca tgatcgcatt ttgtcggcat tcatcgtttg gatagttcag ctcaaggcat
14701 tcactgattg atgccacaag acacagaatg ccagacgcat ccgttgtggt gatagtcatc
14761 ccgcgcgatg ccatctgact aaccagttct tcaggtgttg gtgctgtcat ttctctttga
14821 ctcccggact ttccaccaca tttcaaacag attctttatt accagagaca gagcgccgag
14881 gattgacgct actgccgccc actccgtcag gctgtgtgga atcatagcct caaaataaga
14941 ctgcgtgaca ggcgtttgct ctgcaacttt caggccaatg ccagtaccaa tagaggcata
15001 tccagccttg tcaatcacct gtcctgtcgt tccgcttata atctgctctg cgacttgcct
15061 tagtgctccg ttcattgtgt gtctcgctga tgatatgttt ccagcactta tagatttgaa
15121 tcagcgaaaa aacaatggcg accacgccaa gaataatgtc caatttcgcc gccccattta
15181 aaagtgatgg aaaggatgaa cagatgggtg ccgataataa taaatgcgta ctgcgcatga
15241 agcggcgttt ctacaggcgt gataaattcc catacgaatg actctatcgc taccagccat
15301 tcgtaaaggc tcatcgtcag cacgcagaga gccatctttg tacttttgcg cagtgcaata
15361 gcctggaaca accagaccat agactgcgca aggtaataca gatattcggc gggaaatgaa
15421 tcaacaagca cccatccaag atacacagac atcaccattg ccggaatgaa caccagaaac
15481 gcaaccatgc ccgtacaggc gaaacccagc acatacatga tcatgatggc aacgtctgcg

15541 ccgaacatta tttcttaccg cgcgatggtg agctggtgga accgtttggc ttcactgcgc
15601 cagttttgcc gccagtttta gtattgccag tagcgcgtga acgagacggc gaattggttg
15661 aacccatgtt taaatctcct gttgtttgat tagcatgatt ttagcatatt actgttgacg
15721 tagatttgtg tggagtttat agttagtgac gtagaaacaa caataaatgt tagaggtgag
15781 tgatatgaaa gatgaattta aaggcacaaa aggtcagtgg accaatcatt ctttatttac
15841 tggtgaagcg cgtacatcaa tttggtctgg tgatatttat gtagcagaag taatgagtgt
15901 tagggagtca gaatcggtga agaaggctaa ctcaaatatt atcgctgcag cgccagaatt
15961 actcgaagca cttcgccagc ttcgcgatta tgttgaggat gtttgcgcag tatcgtctga
16021 tgattgccat gaggaacacc cattaaatct ggcaaacaaa gcaatcaaga aagcattagg
16081 aaagaagcaa tgaaaacact aagcaaaatc tattcagaca aagaaacgcg caacggtatc
16141 gcagtcaaca aaacctacct cgtgccagtg gagcaaatct atctggagcc gggatacaac
16201 atccgtgaag cagatgagca gcacgttgaa tatttcgcac agtgctggga atcaggtcag
16261 ccactgccag cattaacagt tattcctgac gagaagggga tacgcattct tgatggtcag
16321 catcgctatc tcggcgcatt gcgtgccatt gagcgtggcg caccaatcgc tcgtattgag
16381 tgcaaggatt tcactggcga tgaggcggac aaaattgcct tcatggtgtc atccagtcag
16441 ggtaagcagc ttgacccgtt tgaacgcgca aaggcttaca cacgactgaa aggctttggc
16501 tggactaacg aggaaatcgc caagaaggta ggtcgctcag tatctgacgt gcaaatgcac
16561 ctgtcgctgg gtgatgttcc ggccgaagta aaagcacgca tcagtgcagg acaaatcagc
16621 tatgcaaacg ccgtagcggt aacgcgtgag catggcgacg atgcggttaa agtcatcgac
16681 gaggcagtag aagaagccaa agcacaaggt aaggacaagg tcacagcgaa ggtgctgaag
16741 tcgaaaaaga ttaagccagt agaccgcctg attgagctgt tgaagcaagc agatcacgtg
16801 attctccctg ctggtcattt tgtggcagag gatgaggaat ttatccagat tcctgttgct
16861 gatattcacg aggtcatggc aattctggag aagatgtgat gaatgagcat gatgataaat
16921 taattgcagg atggggcgac ccattactgg ctaagaaagt tcactatttc cgtgatggga
16981 agattatcag tgaatgtgga aagtggatgt ttggcggtga aagagaagat atatcagcat
17041 ggaagcatga cgcaatatgc aaacagtgtc tcaagaagta cgagaagatg gagaaaatgt
17101 gatgagcaag tatgaaaaac ttgattcgat gattatggat attcttgatt tcaaaattcc
17161 gacgccattc atggcaattc atttttctga tggcatccat agtggcatat acgcagagtg
17221 tgaaaagctg gcaacaaaac cacatgaggg atttcgagtc cttgatcgcc gacttcaggc
17281 gttgcgaaag aaaggtctca tcttatcgca tggagcaagt aaaggatggg tgaagataaa
17341 tcatgaacgc tgaacaattc atcgaaaaac aacttcgcgc caagctgcct gacatcgacc
17401 agatggcaat tgatgcggcg attcagtact acaagcgcaa tcagagcgct aagaagggcg
17461 gcatttttga agaatgcctg aaggttgcaa aacagcacat gattaaggtg aagtgatgaa
17521 actaaaaatc agcaaattat tacttgaatc agcattaatc tttcaggcgc gcaatgatgt
17581 gcgatattac ctgaatggaa tctgctttat gcctgatggt cgcattgcct caactgacgg
17641 tcatcgtgcg tttattggtg gaaaccatga taataatctg acagaaaatg tgattatcaa
17701 gattggcaag tcaccaacaa agcgttatga gcacgccatc attgatacaa agtcgaaaat
17761 tgcaacgtat cataatgaag ctggcgtgat ggttggcgct ggtatctgcg aagagattga
17821 tggtcgattc cctgacattg accgcgtgat accaaaagaa acaaaagcag cagatgaaat
17881 tggcttcaat gctggctatc tggttgatat cgagaaagct gcgaagctgt ttaatcctaa
17941 attctgtggc gtcaaatttg aactgaaggg aaatacaaat gccgcagttt gctgccttag
18001 cgcgccatct ggcgagactg cgaagattgt tgttatgccg atgcgcctgt agcaataaaa
18061 gcccctttac ggggcttttc ttttatcacg ccagataagt atcatcctca aaccacgaga
18121 catacgcatt gatattctgc accgcagtat ccagtgatgt tatgcgaagc agatatgtgg

18181 tatttggtgc catgattatt tgctcaccca atcgagcctg cgagttaccc tgcccctgat
18241 tcgatgcatt cccttcgcta tacgttgctg caactgtcag ctgtccaatg tttgtcactg
18301 tagagccagt cagcagctgt gccgtagctg cctgaggcgt tatgtcgttg ggattattaa
18361 tctccgcagc agtaccgcca gcggcaaccg cgccacgata gattgatgct acgacacctt
18421 tccctgtgta accaataatc cgctggttaa atatcacctg ttttgttccg gtgataaaaa
18481 cgctgtcaag gtttgagccg ccagcaacat cagtcaccct gcgcgatgca gtaaacagct
18541 tgccttgctt attggcgcgc tcaatatatg actgtgtggt aatgtcgctt aacgccacat
18601 caggctgaag gtttactacc gattgaggag acacgccagc gcgccacacc gaaacgcgca
18661 gcgaacttac tgctgctggc aattccgttt tgattatctt caggcgcagc gctacgccat
18721 aataattatt attgttgacg tcaatccagt agttgccaga ttcaaatggc gacactttca
18781 ctgatacggt gccactgaca agtggtgtgt atgcgccaga tgaattaatc ggcatcacgt
18841 taacctgtag tgccgtccag tcagcagaca tcagctcatc aagtatcacc tcgccatctt
18901 cgcgcgtggt gaatatgtcg tatcttacag ccatagaaac ccccaataaa aaacccgcat
18961 tgtgcgggtt cagtttactc tgctttcttt ctctttgtcg tcttttcttg cggggttgcg
19021 acttcgaaag acttttccag ttctggcatg atgcgcagct ttggtagcag atgctcgtct
19081 ggttcggtaa tcacctcgcc aagctgcaac tcgcgaatct tgcctttctc cttaacaaag
19141 attccgcgtg cgatgacttc gtatttagcc attattcacc tcaaattcat aaaggggctt
19201 tcgccccttt ttttattaca ccggagtttg cgtgccgtag ccgttgaaca ctttggactt
19261 gccagtgaaa tccttgcgca cctgaagccc catagctgac cacacgagga agttgaagtt
19321 atcgtgcggg ttgacacggg ctgccgcata ggtggaaaca ggctgggcaa cgcgcggacg
19381 gatgtacatg tcgttgcgaa catagccaac aaagtggtta ccagtcagca ggaagttggt
19441 gccaatcttg ccaatgcgac cgttgccgaa ctgcgtgatg tactgctcaa ccgtgccgcc
19501 tttgaatcct gctgcattag aatacggacg catgaagcta cggcgcactg ccggggagac
19561 ccacagagtc acctgctcaa atacgttctg cgcatccaga atagcctgga aatcctgatt
19621 gaagaaggtc actatttcgt ctggtgttgc agtttgcagg tcgatattca ggccgccaga
19681 agcattcagg ttaacctgaa cagtgttcgg gtggttggtg ataccgtagc cggtgtaaac
19741 gccgttcacg ttcagagtct ggtcgccagt cagcaggtat tgcgccatat cggaacgcag
19801 gttaaaggtg acgttggcct gatcgtccag cagtgggtaa aaaccttcag actgcatacc
19861 aagcagttcg cgccattcgc ggctgtagcc agtcttgaag attggaatca catcgccagt
19921 gtaatcgtag cgagttttat ccaaatcttc cggctcctga ccagacagag tgcgaacaac
19981 cttgccagca tcagaagcaa tgcggctgat tgccacagtc ttaccaatgt tgatgtttgc
20041 cgcgataccc atcaggtcag ccatcatgtc ctgaccggct tcattgcgga aaacgcgggt
20101 ggtaacgtta tccacgtcgc gccagtaatc tttcgtcacc agtgcggtgg cgttaactcc
20161 ataggttttc gccagttcag cttctgcatt gcagaacacc ttgcggtcga tggtgagatg
20221 tttccactga tcagccacca ctgcggagtt ggctaccagg tctttggtaa aaataatctt
20281 ttccattatt aagctccagc aggcatggaa gcattgccag cacgacgaac tgcaaccagc
20341 tcagcgccat cagaggcaac ggtgtaagtt tcataagaat agaacaggat gttgtcacca
20401 tcaccagcaa ccttcagcgc accagcgcca ttgcttgcca gcggagtacc tttcttcagc
20461 actgaactct gcgcaaccag tgcgtgatag gttacgccaa attcacactg cacagccata
20521 ccagtagcat tagccggaac cgcttcagac acatcaccac caccgaggta gttgtgctga
20581 agcacgtaag ggaaaccctg accgccagcg gtagcatgag cgattatttt gtcgtcatcg
20641 ttaaaagtta ccagtgcgcc cggttgcaat gaggcattca tgatgccttc acgaatctgc
20701 gggtcgttct tgcgagccgg gccaccaatg atggtgccat aacggatagt agccattatt
20761 ctggtgcctc catatcaaaa tcgtcatcag cgtggtttgg ctggaagcca cctttcagcg

20821 cggcaggctt gctggtcagc gcataggttt cacgcagtgc ttcgcctttc agtgcattca
20881 cagcggattc cggcagtttc agttcagcga tgatagcagc acgcatcgcg gtttcttcct
20941 gctcggtatt cgcctgtaat tgctctttca gcttaacgtt ttcagcttcg atgtcggtca
21001 gcttctggtt gacagctgtc aacgattcct gaaccggctt gagggcttca gcgagtaccg
21061 cctgtaattc ctcgttcgtc attgagattt ccccttcagt tgattttacc ggctcaagtt
21121 cagtcttata aacagccttg acccgctcac cgaccaattt taccacatca tcttcaacga
21181 tgtagaactg ctgaaaaatc tggcctttga tttcaaatcc gacgcggtcg tcgtacacgg
21241 ccacaatgta aggccacaca tcatcaccga cttcagcttt cagaatctgg cgaatctgct
21301 cactgatgtt ctcaaacgac aggtctgatt tgttggtgat gtagttaatg accttgtgca
21361 gccacgattt ataattaatc ttgttggcgc tttcgtcagg cacggttgaa tcctcaaggt
21421 ttacggtgat gcgttcgatt tgctcgccat tggtcgcaaa gattcccaca ccatcttctg
21481 gcgttccggc tcccggcact cccggtggca ggatggcgag atgatcccac tccatgttgc
21541 gtgcaatcca tgagtatttc ttacctttgc ttgtaccttc cgcagcttcg cgattgagca
21601 ataagccagt ggacacatgg attggttcag caccctcagc agagttcttt aacgcctcaa
21661 tacgcccaat taactcctga ccgtgctcag ttctctcggc aacggcaata ttgatgtaca
21721 ggtccaccaa tgccttgctg ccgtcatgag atgagttttc aatccaagcg ccaacgctga
21781 actggttagc ggcgcgggtc atgcttgcgg agacgtattt gccgtcgatc atcgggtgat
21841 tgtatggtgc aggcttgcca tcaagtccgt gatagctctt gcggatttcc tcgcccggat
21901 acaggccgtc attcatcaca acatcatcaa ccactggcac gacgttttta atcacgtagt
21961 gcgggtgccc atcaatgatt ttctcactga tattgctggc tgaattgatg gtcgtcagga
22021 cgttaacctg caatttatta ttcatgtgct taaacgcttc cacttctgcg aggcgttttt
22081 tggctacctc ttctgtgtca tattcgccaa attgctgtga gccgtctttc gacttgacca
22141 cccacttatc gccaatcttg acaatcatgg ttaactctcc acggttagtt tgtggtcgaa
22201 ttataacaca gggagatata gcaccatgag gcggtagcca gcgcaaataa tggcgaattg
22261 gtgagtgcgt atagcgtgag aaagtaagtg atagggatta tgttcatggg ctacctcctg
22321 attaaatgat agcagcccat gatgaacaaa agatatacgg agttagtctg aaatcacatc
22381 atccataaat gcaagtgcag cacctgacag gcagaacaga gcgccatagc aaatcatctg
22441 gtacagagtg tcagcctcaa ataccсttgc gaatgcgtag gctgataata tccaaagcaa
22501 tggaatcatg gtctacccct gttttcttta tatagagaat cgcaaatctt acggtacgca
22561 atgccaaaga agtcgtattt ctcattatat cgaccaccta ccgcgctaat ttgatttgct
22621 gcaatggtgc ttgctgcatt aatcaatgca acctgaacct caacaggcag ttctggaaat
22681 tttggctcgc tcataaccca gcctcctgtt ttgcgcggtg cacgtatgac ataaacttgc
22741 caatcggcat ttccttgcgt atttgcgcca gaatagctcc gtgaatcatc ctttcctcgc
22801 caaaataaag tttatccaga cgagttttaa taagtgctcg cttgcgcttc atgtggtcgc
22861 gcgccttaag tgctttctca tgccagacgc ggtcgttctt cttatccgca tattgcagtt
22921 gacgatcaac ggtttcgatt tcaaacgcca gttgcacgtc ataatcttca agctgaatga
22981 tgtctgcttt cataatgtcg tttagttgga taatcatttt ttcacctcta atcctgcttg
23041 ctcaattacc atttcatatg catccattgc gtcaccaaat ccattggagt aatcaaccgt
23101 gtagccttta tttaatgccg atcttgaatc aatgaaatca ggagactcaa tctcaacttc
23161 agcgcgcgat gcctgccatg ctttccatgc ccatgcagtt ggcgaataca cataatcaca
23221 gtcaccataa acaaggctca ttccgttctc tttggcccac ccttcaaact gctttctgct
23281 cgtcatcttc atctcctcca ctatccagcc catctttcta cggttattgg caataaactt
23341 atccgatgtt acaaaaagag tcctgccaga tttatgatta atagcccatc tcatttagta
23401 catcctcagc aagtttgcgg aacataattt gcactcttgc cactttgcgc cactcagcct

23461 cggtgagaat cacatcctca tgctgcggca ttcccgacat ttcgcatggt ggcaatggct
23521 catactcttg cttcttgcgt ttggcttttc ccatgatgac ctcttgcgct gcgaagaagt
23581 gaattaaaca ggttgctcac atttacttcc tgctcccgcc atgagtaacg ccacaacttc
23641 ttgtggtggt cataacttct cgtcacatcg ccgttgtgaa acatgatgcg gatgcggctt
23701 ttcactattc ggtaatcaat gccagtggct acgctgattt ctttcacttc cgcaccagag
23761 ttatcaagca aatggcactt aatcgcacca tcaataccag cggaatcatc agagataaaa
23821 tatttatact gccacttccc gccgcgaatc actgacttct cacggcgaat aaagccaagc
23881 gcaagcatct catgtaagcg atgtgttgtg gtgcttgaat gcttaccgcc gcaatgcttt
23941 tcaatatatg cacgcgttgc tcctgggtga ttcataatca cgcgcatgat ttgtgattta
24001 taatccataa tcacgctcct gtgctgcctg attgaagtcg tcggctgtgt agaggtgtcc
24061 gcctcttgtg ttccatgact tcactgctga tgcttggtga ggccactcat cagtaccaca
24121 cccacactgc gagcaagcga cataaaacgc tccaggacca tcgttatcta tatacgcatg
24181 actatcccca caaaacggac actcaagcag catttcatca ttcgtcatcg gcatatgtgg
24241 tgcgctcatt tcaaccattc tccaagatta ttaaatttag gtgcatcgcc agaccagtcg
24301 ataacgtctt tctgctgact gcgcttgcgt tgcaggcggt ttcgcacttc ccgcagctca
24361 cattcaagcc attcgcgagt gcgttcaact tcattcagtc gcttgattaa cgattcctcg
24421 tacagctcgt catgcgccat ttataatttc tcccgtagaa tttgataaca acgtgctcaa
24481 cgcacagata accttcatcc tcaaccgtta ctggttgaac tataaaccac cgcagatata
24541 agttcgtcag ccttttcacg acctgcaacc tccgcttcaa gcgccagtga acacatggca
24601 gtttccacaa acttaatggt atcttcgcga acgttaagaa tctccataac ctcgcgaact
24661 tctttctggt atctcgctgc tatttctttg tatcgcattt ccacatcacc ttttccgttg
24721 tggtacaatc tacgtcaata agtattgact aagtgatgta gaaacgtcaa gatgtttttc
24781 gagaaggagt agtaaatatg ccaagaccac gacgcgagcc gatggacatt atcaccagca
24841 ttgttgagaa gcggcagccg ctgacactcc gtgatgttcg ctactttgcc cgttgctatg
24901 tggcgctggc tgatatgcca aaggatgata tgtaccagat gattcgtgag aattttaatg
24961 ttgatgaaaa taaccgggtg acgatgaaat gaaaaaatgg aaatatctga aaggttatga
25021 ggatgatttt gttgaccatg acacggcttg tcttgtagtt aagtcaggaa gtactggtga
25081 gattttctat ctaagcatag attacgctgg tcgcattgaa gtgattgagg gttgcgggga
25141 tgttgttatt gcataccgcg agccaattac cgacgaacaa gacctcaacg attgcattgg
25201 agcgccggaa gctgacgcaa cagagcaact catcaccgaa cgcggcagtc gatacggcaa
25261 attcaaagac ggcgcagcca tcatgcagga gttgaaattc gtcatgcgcg aggttgacgg
25321 gtggcataac ctcacaccaa gccaacgcga ggcgctcgac atgattcagc acaagattgg
25381 gcgtattctg aatggtgacc cgacttatga tgatagctgg aaagacattg ctggctatgc
25441 gacattaatt gttaatgaac tgaacggaga gattaaataa tgtcattctg cgacatcaca
25501 atcgcgcaac gaaacgcgaa ctttaccaat attgctgaca cgtccgcaca actggtgtca
25561 ctgaacagcg acggtagcgc agtgctgaaa atcggcactg aaacagcgca attcatcgtg
25621 cagaatctgt cacaggcgaa cgcaaagcag gtgctgatta gcactggtag cgttctgttt
25681 ctggctggta attacaacgc accaaatctt gagtgctcac tggtgcgcat cgtcgaaaca
25741 actgcggagg agtctgttga tgatccaaca acactgccag cagagtgagc caccaaaacc
25801 atcagcatgg tgcgagaaaa tggagcgcaa tgcgaaagat ggcgatgaag cgtatgttta
25861 tttccagcta aagcagatgt ggaagcaacg agaaaactca gagcaaacca attaaacaca
25921 agccccattg cggggctttt tcatccctta aactttatcc agccgaacat cctgacacca
25981 agccacataa cgccagttct cagtgatgaa acaccgctat cctttaatgc ctgtctgaat
26041 gggtggcata cagatagtca tgaacggctg ctgctttctg accaacatca ccaataaaag

26101 cgaacaggta aggaagccga ggaacactgg caaaatcagt gcgaaatcct gctggcactt
26161 ttatttcttg atgcctgaac tcatagattg gcggctcaat cagtgtccac gttgagtcct
26221 tgttcagctt gacgataaga tcggatttga acatttattt tttatctcct tgtcagtgat
26281 tcgtattgct tctggcatgc taatcccgcc tctcttgctc tgtcagcgta atctgccagt
26341 tgtcgatttc tttcgacaga ttctgagagc agctgggcaa gcaaaactcc ggtttctgcg
26401 gctggattgc caatggactc agcggtggaa tatccgacga gttgcttgcg gatgtttgca
26461 agttgttgct gcaacctgcc agacttagcg gcagcattaa cagcatcatt gcgcgcagca
26521 tcaatccttt gctgtgcgtt agcctgaatc ttttgcagtt ctgcattgcg tcgttgctcc
26581 tctttttcgt ctgcggcctt ctgttctgca attgcttgag ctttgccttc tgcgtatcgc
26641 tggtcgccgt actctacaac ctctccgcga atccatagcg caccagcagc aacaattata
26701 ataattgcca gtggttgcca gtatttagcg agaattgaca tcattgcgca gcctccggtt
26761 ttccttcctc atgccgtgca ttttgccgag gatgccaacc agcatgattg agtagctcac
26821 ccctttaacc acgattggcg gcagcgctgc tttcaggtcg tccggcatca ttacccacac
26881 atgcatcatt gcgtcaggcc atagctgcaa tagcgaacag aatgaaatcc acgcgccgag
26941 cagccagttg cttagttttt tcatgccaca actccgccag cctctttgta tacctgaatc
27001 aacttatcca gtttctgctc atgctgacca tatccagcac cgggcaatga agcccaacgt
27061 gagcggcatt tatgaatggc atcagcaatg cggccagcct caacatcggc ggtggctttg
27121 cattcgcgga ttagctgcat tgcaatagca tcctgcgatg cgggggagaa gtccggtaaa
27181 cgcaactgct ttttgtacgc atcataaaat ttagccagca cctgatagcg cccagcggca
27241 gtggatttga tgcccagctt aggcaggcta atcagcttgc gcgggtgatc ggaatattcg
27301 gtaaacagtg agccacctac aatcacatca tagccatgat tgttggtttt ctgtcgcccg
27361 ttatccgtgc cctcgctgta cgccagcata tccagaaacg ccttcatgtt tttgctaata
27421 tccataccag taaacctctt tttcagcttt acgttttgcc ttgtcgccag tcttctcgcc
27481 ccacaagatg aaatgagcaa cggcacacga gaagcagcgt agattgtgct tcttcagaag
27541 cgttgatttg cggaaagtgt caatgccgat gtcggtggcg aggcttgtca gggcatcaaa
27601 ctgattctgc gttgtctcgg cggtgatgta aggcgatata tccacggcat cggtgacatc
27661 aagcgcctcc atgccgcgtt gcgatagttc cattctgcct cctgttcaaa taatgattaa
27721 ttctatcaca acgactattg acgtagattg agtggtggtg catagtatct acatcaaatg
27781 gtgagtgagg tggtggatat gtgtgattgc atgaacaaaa tggaagagtt actgaaggaa
27841 agattgatgg agcgcgtgcc aagcggttcg gaggtgagtt ctaatgtttt tgacaaaact
27901 ggttgggata atcaattcat tagcctttcg tcaggaaagg tttttgtgat gctcaaatac
27961 aggctagcat acagggcaag aaagaaaaac ggagaactgg ctaaaaactt aacaagattg
28021 gagtctaacg taaaaatgtc atactgccca ttttgtggcg agaagcaggt ggattaaaca
28081 tggtaatggt caaattcaaa gaaaacggac gctgcggagt attcggtctt aagcaaatca
28141 aaatccgtcc gtgcggcaag gtgatcgcgc catttggtct tgtgcagatg cgtgaagttg
28201 agattgttga atatatcaag tgaggttggt gatggaagaa tttaaaggca cgcatggccc
28261 gtggaattac tgtgctgatg agccggattg ggttacagat agcaatgaca atatagcggt
28321 agcaagggtc actcgttaca acgcggatgc ggaagctcaa catgcaaacg caaagctgat
28381 agcagcatct cctgacctgc tctcagcact ccagcaacta ctggaaatct acgacgataa
28441 ctccggaaaa gtctggacaa catcaagcaa gcgtcgcgct ctggataatg cgcgcgaggc
28501 ggttaataaa gcgttgggag aataattatg tggcttgtat ttaatattgg atgaatcgaa
28561 tgcggtgttt catcaaatgt tgttgctgtt tatccaactg aacaggaggc taatgcggtg
28621 gctaaaaaac tgaatgaggt tcactcatgg cgagaaggag gccaaaactc gtatgaggta
28681 tttaagctgg gtgatctgta cgaggttgag ggtgaatata aagaaatact ttctgatgga

28741 gaaacaaaat gaaacttatt gacctgttag ttaaagaatt gcctaagcgt ggtgggtggc
28801 ctcaaaactc gctgtcaatt actcaagata atgacggttc actgtgcgta tgggacacga
28861 atgacccaca ctacgaaggg ttctcatgga aacaccacac tggcaatagt cttatgcatt
28921 tctggtgcga agaagcggcc atgccattat cttccgacca caaggagtca atagtaacct
28981 actggcaata caaagcagca ctcgccgcat cgcaaaagcc aacatgggac ggcgaaggtc
29041 ttccgccagt tggtgcaaaa gtagaattct tcattaatcc aaagttcgga taccgcaatg
29101 cctggattcc tgatgctggc actgaaatgg aagttgtagc gcacaaaaca acaactgacg
29161 ggaacgatgt tgctgtttgc tattgggatg atggtggcgc tggtcggtca tgctgcttca
29221 taccggaaag cctaaaacca ctacgcacag aagcagaaag gaggcgcgac gcatttataa
29281 atgcagttct tgatggtatg cgcgtaatcc cttgtgattt gtcgttgcgt gatgaggttg
29341 ctgttattta cgacgccatc gcagcaggaa aaattcccgg cgtgaaactg gatgattaaa
29401 acaatattca gcgtcgtaga gatattgtgc tatgcgataa taatatttgt tctgtgcatg
29461 ctgataagat aaagcaaagc cctcacttag agggcttttt gtttgctgat tgccacgctt
29521 ctcgctgctt atcaagcctt tcctgcgacg attccagaat aactggcttg ccatccatta
29581 ataacgcagg ggtttgtgca caatggcaat tatatctatt gccattctcg ctataaaacg
29641 tgtcaatctc ttctggcgtg taaaaccgtc cgtgacgagc ggcgtgcgtc tggcgagttg
29701 tacgcattaa tgcagactgc cacagcatca ccgtctcaat gcctaattct tcgcgcgcct
29761 caatgacctc tcgcctgttt gcctgccgaa gtgttccggt gatttctgtc tgcgcaatct
29821 gttttgcgta gctgtgggaa acgtcaactc gcttaacaat gtcagcttca acatcgcgag
29881 gattagcgcc acgggcaatg ccttccataa tgacagacgc cagttgctga cgggaataat
29941 cactcaatcc gcgccagtct gagtaacctt gcgtataagc cagttgcagt cgattcagat
30001 aaggctcact gtagagtatc gcggaaatcg gcctttgctc ggcgtaaacc ggagacaggc
30061 ttgacagttc agagttggcc ttctgcgtgc ctgcttgata cgcatcaccg atgaatacat
30121 tggcccacat cctgccgtga tcaagatcat caccttcaag aagaatctcg tcaataagtg
30181 cctgcaactc atccatgaaa gtggcagccc gcgcactgct gaaatcgtag aaatacagac
30241 cgcttgattc tgcgttggtc tgaccgttcg gaatggtgcg aaacagctca agtgcacgag
30301 tcctgagctg tttgtatttg cgcgttatca ccttatccat cttcgacagg cgggttgcag
30361 cgcctaacgg gtcggtcaga ctctgcgata tgcgcggttg tggtagtctt gcgttaaacc
30421 ggagtatctt cattttcatc ttccggcggt gtgtcttcgc gatagctttc gtcaagttca
30481 atcggctcca taccaaccat tccgcgagcc tcatcgacag tcagcaacgc agattgacca
30541 gcatcaaaga acgacttgtt cgcagtggcg agcttggaca gcaactccgc tttatccagt
30601 tctgaaggcg caagcaggtc atcccactta accttgtagc catttgccgg ggctttatcg
30661 acaacaccaa actgaatcat gcgctcaacg aacattgaga tgacataatc aacccatgtt
30721 tcgcggcgct gcttggctgt cattgcctcc tgcatcttgt cttcatcgct cgcaaggcgt
30781 ccggtttgct ggccaaaaat gacggtgaac ggaagcgcca ttgatgcagc gaactggttg
30841 gcagcaactt cccatgtggg ttttgggtcg gctggcgtta cagcaagaac cttagcatca
30901 gcccccattg tgaacatagc tgcgtcaatc ccggagttca gtgcctcaat gttctcattc
30961 atgatgtcgg tgagttcttc gatatcgaca cccattgact gcgcaaggct tgctggtgtt
31021 acgttatcct tcgtgtagtt taccgccagt tgacggctgg cattcttcag gaacccttcc
31081 gctgagctac cggaaacctt agccatgtcg ataaggctat tgtaaccagc gcgcagcatt
31141 gggataccac tgaacatgct gccgtcaaaa ctaccctcag ccagaatgat gatgcggtca
31201 ggatgaatct gcactgaacg atcaggctta ccgtcgctgt cgaagtcttc cacggcgctt
31261 tcctgatatt cgtacatctc aggcattccg tagtcttcgc tggtttcatc gttattccac
31321 gcactgactc gtagttgctc ctcccacaca ggaataaagc gaacgatgga tttatcttta

31381 atacgtctgg ttttggtgat gtcaacaggc tcgctccact ggcgaccatc gcggatttgc
31441 aggataacgg cggaatagcg gttgatggcg ttacgcttgt cagcctcctt gataaacgga
31501 taggctcgct tcatcatgtc gttgatggac aattcccacg gcgttgaatt cttgtcatcc
31561 tcgccatctt cgacaacttc agggtatttc tgccagcatt tattaataat acggttaatg
31621 ccagcggctg cggctgggtg tcgctcatag gcgtagcgga acatctcggc ggtgatctcc
31681 tgcgggtagc cgcattccgt ccagagtctg tcgtgcttct ggtcgagatt cttcccacca
31741 gcacaaagcc gttgctgctg aagcgcccgg ttattgttcg ccacgcggtc gcgtatatag
31801 gcgtttaatg catcaatttt ggacatatgt caccgataaa aaatccctca cgatggaggg
31861 attatagcat gtcacttgtt gcgatttcgg tttctgtatg ccggatagaa ttttgacaga
31921 ggccacaccc agaatttcca aagcatctca tcgtagcttg gtagtgcctc atggaatcgc
31981 ttgccggctg cgtatccatc agggtctcct tcatagaaga actttataaa cccaattctc
32041 gcgtcagcga ccgcgctatt tctaataaac atataagcaa agaacgccat aacaacaaag
32101 ccgataataa tagtcaccat agtcacacct ctatttgaaa tactgataca gcaagcatgg
32161 cacaacacca gtcaccacag cgaaaaatga accaactata aagtgaatcc atctcggatc
32221 attcacttct gcagccatag cccaccagaa cataaagttt agtggaatcc agaacgacca
32281 gaatagtgcc atcactccac cttttcgaat ttgtcgatta ctactttgat gccttgagat
32341 gttagagatg acaccatatc attacgccag aaattctcta gagcatcaac tgcgttggtg
32401 gcaaccatca ctccacttaa cttttctgcc gcacagtcat tattaacaaa gacttccgca
32461 tgaataaaat aaacactcat cactcaatct catcaccatt tacccagctt tgcaggactt
32521 cgataagctg cgcggcttgt tttttatcaa gctgaaccca gtcgtcgcat tgccttaaat
32581 ttattactag ttctgtattt atatacagac tgtggcattc atttgttttt tcaataatca
32641 tcactcacat caacaaagtt caccatcagt taccattaat aacttctgcc atttggctac
32701 tggcttgcac agttcagcct ttagcctgct taacgaacct gtaatatcac tccattcagt
32761 gaacggaacg tatcgcacaa accggtaatc agcaccagac cagtgccagc cgagcgtgta
32821 atcaacataa gagccgtctg actgcatgtt gatgtaatga gctgttgcgc catcatcatt
32881 aatcataacc acctcaacca ctgcgcacga accaccatcg cgagccttgt ttactgcatt
32941 cagatgacag gcatgattgt acagtccggc gccgtcgatg aatgatatat caatcttttt
33001 ggtgtatttc ttgtccgcag cgtcgcggat tattttcttt aatttatttc tgaacatgat
33061 taatccacca tgcggcaatg tcatcaccgg caaatcttga ccacatagtc aatcttgttt
33121 gcattaaaat ggacatcttt aaatgcagcc tctacacttt gataagtgcc attatttctg
33181 cgaacgccca acttcttggc aataaccatt gatttaccag tctctggcac agcaatccaa
33241 ttgccacaat caattagcac atcacagcca aataccaatc tgtgacgggc tttgcgatga
33301 acatgcaatc caccaatgcg cttcggtata atctgacctt ttttaaatag ttcgccactc
33361 atcatgttac cctctcaaaa aatcattctt gcagcagata cctttgtacc cgcgcttctc
33421 ttgcaggtta acgaaaacat catccagaac gcgcagcagg aaatcctcgt cgatatcata
33481 ccgacggcaa atcacatcat caggcacgcc agcgcgcgcc agtgaataaa cctgctcttt
33541 ttcctcctgt gtgaatcctg catagctgcg catagtggta tctccggtaa gcctgatgta
33601 gatatactat gcgctcggtg tagattggtc aagtgtggtg atgcggattt atcaacaata
33661 ataataaatg ttgaagtgga tatagtggtg atgtagaatc atcttatcga caagagatta
33721 aggaacaaaa gaaatgtttg agactaaaaa agaatgcgaa gcgtacattg ccgaaactta
33781 tggtgctgat tacgttaaat ttggcatcgt taccgcccag aaggttggcc caacagtcgc
33841 caagatgctt ggcatcaaag aaggttacta cccatcaaat gcctactaat tacatgccga
33901 gatgccttcg tgaaataccg aaggcgaaag ttaaaccaag aaatcaagcg ataaaagagg
33961 caaaaattga ggctttcaat attgccattt cgataataaa agacagatgc agaaatgaaa

34021 agtctgaacg cattaaatca aacatgtatg cagctgtgag tgatatatca agattgagag
34081 atgagcttta agccctcgaa tgagggcttt attttatcgc ctgcgccgga ttagcattcc
34141 actaccgcgc tgaactatat ggtcattaag tgcgtaacga accgcgtccc aatagtggtt
34201 gtatgcatca acaatatcag tcagcacatt ccctgtcagc ttatcaactt tgtagctgta
34261 cattaccgcc tcattctgca tctccttgca tcgctcatga atgatgatgc tgtcacagcc
34321 acgcagccac gttacaccat cctcaacgct tcccggccat ttcgtgcacg ggtggatatc
34381 gaatccagaa cgcttgatgt gactgatggt ttcaggtcga gcgcagtcgg cataccatcg
34441 tgcgcgctta gccatcggga atgattgctc catcgcagcc ggtgtatcgg taatctcaag
34501 ccccaccttg ccgtactcgc gattaatata aatgttgcgc cgcccacctg gtaactcctc
34561 gatgtaaacc tcaaccatcg cggtagggtc cgtgctgaat ccgaagtcca tcccgaaata
34621 tgggccatgc cattcaggtt tgacctcgaa gttatcaatt cgccacttgc cgccgaagac
34681 ctgttcgtcg ctacgcttgt tgaacttgcc ttcccatatc cacatgtagc ggtcgaaatc
34741 gacgcgcttc atttgctcca tagcggatgg caacggcgta tcccagaacc acgggttgtc
34801 tgaatagtta cattcgataa tcagaatttc atcattttcg aagatgccat caaccatctg
34861 tgagtgatag ggggcaatcc agtttttcca tgtggggtct gtttccttgt ttgggttgaa
34921 tgagcaccac agttcggaat tggcagcacg gatggtaggc acaagaatat cccagcttgt
34981 ctggctgacg ttctctgctt cctctaccca accgacggta atcccagcaa aacccttaac
35041 tgtagtctga ttgcgataca accccttaaa gcgaaatttg gccttagtct ttttgtgtgt
35101 tatctcgttg ttgataacgc gaaactctgc cgattcaccc ttgcgggcta tctcatcaac
35161 aagctcctga tagcttgagt cttcaatgga ttgctgaatc tcacgaaaac aggcaaagcg
35221 gtcagggcga aaccttgcgc gctcagtgag tatagtgata atcgttcttg tcttgcctga
35281 cccgcggcca ccgtagataa acttgaatcg ttttgggtaa agcaggcgct ccagttttgc
35341 gggaatcagg tggtcagcat gagtggttgc atttgtcacg tcatcaacgc cagtggcggt
35401 catccttagc cgtttgatga cgtttttctg tatgtcgcag atgccaaaaa tggctgactc
35461 ggcgacatct gtcatttcat catcaatctg cgcttccagc ttttcgattg ccagcgcgga
35521 gaggcgttta cgagccattg gctagcccct gaaggatagt tgccacctct tcatcgctca
35581 ttgctttgtc aaatgtaacg ctgcgagcac tgctgttgcg tggcaatagc acctcatcac
35641 attttgattt atagttattt gcttcatcat ccattctgct gctccagcaa tttctccagt
35701 cgctcaaggc gcgcggcgag ttcggtaact tcggcaatat ccagtcccgt cctgataacc
35761 tcagcaaacg ttttaccaat gtccacaggt ataacgccag cggctatgcc acgaataaca
35821 gcatcaatct tctcgactgg agttccgtca tcagggaaat ccacctcaaa cactggtgct
35881 acaggcttag gcagcggact gaatcgaacg ataagctcct tcatcattga cgtgtcacgc
35941 tcaatcatcg ccatctcaac gatggtgtca tagagctttt cctcactaaa ccccttccgc
36001 tcaagagctt caacgagcaa cttgcgctta tcttttccgc gtttattttg tggttgattt
36061 tctttactga atagcttttt agccatagat aaaacctatc aatattccgc ttttttccg
36121 ttattcgata ttgtaacaga ttctacacca tgacgttaat ggcaaaaaaa gaacccgcac
36181 aaggcgggtt aaaggttggg tgatgatatg agtgaagcaa tggtggttat gcgtttattg
36241 tacaccatca ctatcacgct tgccagcata gcaccatgcg ttttctgttt gcgtctgcac
36301 atgaacgatt tcaagcgcat cagggaatgt ctttgccacc ttagcgatga aatcgtccag
36361 ttcgtgctgt ttcgcgagtt gccaaacttt ctgtttagtg gtttgcatta gcataatctc
36421 tcgttgagca catgcactta atgttatgca cgccgttatc aggcacaacc tcaatgcgat
36481 tgccgagctg caccatcatg taatgacagg tcatcatgca atcccagtag cgtttccaca
36541 taaattccat tgcggcgttg atgtcgtcaa acataatgca caactcctgc aatagctgct
36601 accagcgcca aaacataaac agcaaggaaa attttagcgc cagtggtgta tcggcgtcgt

36661 tggtgtttgg tcatctcaat atctccacta aagcgtgcgc cacatagagc gcactgataa
36721 ttaatatgaa cgggtactat tcacttttag ccaggctttc gaattgctga gcgcgatgtc
36781 aaactgaaac ctcttgacaa catcgtcaaa catgacaaat tcgccttcat ggtcttcagt
36841 catgtcagca cagcagttcg gactgaatgt gtcgcaacca ccagtattaa gatcgtatcg
36901 cttgagtttt acgatatttg ataaattaag cgccaataca gcaaggtcat aaacctcttc
36961 ggcggtataa cctgcaccat gtccatacat tttaatgcgc gatattattt cttctactct
37021 ttgttttgtg attgtcattt tttatcaact cctttacgaa gctgggcggc gaactcgtta
37081 agtgatatgt agcaatctcc aaatgttaac gaaccgctcg actgcatatg ctccatagcc
37141 atctccactc cctgcgcccg tacttcagcc aggaaggcgt cggttgctgg tgttttgttg
37201 tcgcacatca attcaatcgc gtcgttccca tcaagattgc agaactccca tgctgacgaa
37261 tagaactcga tgccaggcca gaccaagatt ctattcattt tgtcatttag tgtcgcattc
37321 tccgctgcca acgcagcgca tttagcttcg agttccgcgt agtcctcgta attaacataa
37381 gcaccatcgc cacactcatc catagacatg ctaccaaaag cagaaatgtc ataacgctta
37441 acttcactca taccctaacc ccaataattt cttcataata accatcttct tcaaactcaa
37501 tctttgcgat tgtgagagca tcctcatctg tgatgtcaat tgctttgaat ttgcactttg
37561 ccagcacaat gcagcctgac aggattaacc acttattcca ccagcgcgct cgcttgactg
37621 tcttgcacgc atacattgcg ttccgcacaa accagaactc atatgttttt acttttgcca
37681 tatcatcacc attaaaaata agagagctga atcgatatca gaatacgata atcaactcat
37741 ccattactct tcgcttacct tcaccattaa aaacgcgcac aacgcgattt acgcgaatag
37801 attccattgc attacgtgct ttcttgctca taaagttctg cgcgccaacg tgtccaagtt
37861 cttgttccag cttctcgcga cgatagatag tttttgccat ttatttcgcc tcgcaagagt
37921 tagtgattga cttaatgcga cggcgataca gctgcattat gtgctcgtta tgccagttgc
37981 tcattctgtt actccttata acctgatttg gaaactgcta ctgcgataat gatgagcgcc
38041 acgataacca gactggcgat aatttcaaat gatgcgcctg tcatttgctc aacccatacg
38101 ctgagattaa agcctgcatt gctgcgttcc agaatgcttc agcaggaatg ctgttgtttg
38161 tgcgctcaac tgcaatgcgt gccatagcct gcgcatcttc aaattcttca cggtctttga
38221 ttttcatctc ttcaccctcg tttgttccga tgagttcaat ctacatcaac gatgattcta
38281 cgtcaacagc aagttaccca aataacgcaa aatccggtaa ctcggaacat aaaaaccggt
38341 aactgttaca aatcaattac ttatgttgtt ttataccatt tttgccatta aaaagttacc
38401 ggagttaccg gtcgtccggt aacttgaaac ccgcgaacag caaggctctc aggcgagtta
38461 ccggatttac cgggtaactc cttacatata taaaagggtt gaatttctta ttatatatat
38521 gatttataag cattttattc aattttgata caaattctca tttttgaaat ttttatagac
38581 tcatatgcaa aaaatccggt aactccggta actctatatc tatcagtaac ttgcatcggt
38641 aactgatcgg taaaaacccg gtaactccgg taactactgt aaagatgtgt aagttagaac
38701 gttatcttgc tgtcgttgac gtgtacataa tattcatgta tattttttac atcacaaaag
38761 gaggactgaa tgtacccaga tggcactaaa ttaatgtttg catacgagct gttatcttca
38821 atgaaatctg gagaactggt tggtattcac ttcgagaatg aatactcaag cgaaataatc
38881 aaaggagaaa aggaaaattg ctgttgtgta gtgatcgatg aaaagtaccg catgacgcac
38941 aacatgttca ggtcttactg caccagaata gccaatgaac aaggtatggt catcaaaaca
39001 aaaaccagtg aaagcaaaaa attcatgtat atctggagag tcctctgatg actgattcaa
39061 agcaaatcat tgaactaaag gaatctctta agccaacagg taagcctgat tctggaaatc
39121 cacttgatga aattgacgtg ccagaattcc gtgaaggata caaaattcct cctggctctc
39181 ttggtgaatt catgtgcgag atagagaatc attttgaaaa tcgcgaagaa ggagaggtat
39241 accgattgcc gggtgctatc gcgcttgctc aggtgatggc tggcagatac atcattggcc

39301 caagcgcaaa aaacaagtgt gcgacgggga catttattgt tggtcgatct ggcgcaggta
39361 agggtgctcc atctgatttt gttaaggctt acgcagagaa tcttggaata actcctcgcg
39421 tatcaaaaag caccgtaaca tcattgcgac aaatcaaaga acgactaatt gaggctgatg
39481 gtttcttgct ttatatcgca gatgattgtc ctgaacacct tcaggcttgg tcagatacgc
39541 gctcaccgct tggtgaaaca gcttcatggt tcaggacgtc gattagtggt gactggttcc
39601 ctgagtctcc tgtcgttaca ttgtttcagg agaagctggc tagtgctcaa aacccgaagt
39661 taatactctc tgctgcgcag gctcaggggt ggatgatacc tcgcataggt gaatctgatg
39721 gcgctataga ttatcgcaga cttgcgaaaa tgaaccatga tattggaaga agattaaatc
39781 acgccatgca atgttatgac ctgtgcatta acgagaaggg aatacagaac gtaaggttca
39841 ttcctttcat aactgttacc ccagaacagg ggattgcaac agtaaggaga tgggaaaagg
39901 atggcggcat gggcagatct cttttcatta aaggccatga acacatgcca gaacttaaga
39961 atactcctga catggaaatc aataaaacaa taataaacga atggaaacca agaattccag
40021 gtggattctt taatgtggaa tatgcaaatg atggcgtttc gaaatactat gagatgttgc
40081 gcaggcgcat agataagtca agcaacattc ccggtgtaat tggtagcgtc ggccctcgct
40141 ctgctcagat ggttattgag cttgcgactc tttgcgcttt tgctgactta tcatcgcgca
40201 atggcatgac tccgcagata agggaatgcc atattgaatg ggcgtatgca acagtaatga
40261 acagcatgta cgatctgcgt gattatctgg agggagaagc agagtttgac ggacttgaaa
40321 acactgagtg ggacaacatt gttatgaagg ttaaaaaatg cattgaatca aaggctttcg
40381 ctgaaaagcc ttatatttca gtggtaaaaa acaaactttg tcgcgacaga atcagtaaga
40441 taatttccgc cgccgattca aactcaattc aggttacgcc ggataaattt acttacgagg
40501 tcatcattgc catctctgaa aacaggcatt caccaataga gcttgaccca gaaaatccat
40561 ccaatatccg gctttctggt ggcggttcat ggtctgggtt gcggatgaac tcatcagtca
40621 ggaatatcct gtcatcagca atgaagcgaa tgagatttat gaggaacttg aaatgagaac
40681 aatatcaggc gaactttgcc gctcagaagg tttatggcat ttcaggccgt atggttattc
40741 gacgtggttt ttctggagtg ggattaacaa agtatggatt cgctctaatt accacgtgct
40801 taatgattac tggcacagat tttgtgttgg tcacgttgat atttcaaaga tgatgattaa
40861 gtgagtgtaa ggaaatagat atggaatatc acacaggtaa gtcgctgcga aatgtagact
40921 tctggactga agcgccagag ttcaaggaat aacaaaaacc ctccgattgg agggtttttg
40981 ttatctgatg actgacattt tctctatcaa ctcgccagcg tcataaatct ttctcctgat
41041 tctctttgcc atcctgattg tgtcagcatc atcaggtagc atcaccggaa ggcctgcatt
41101 ctctgcggcg cacagctcgt cgaatttata attaactatg cagcgtaacc gtgctttcgt
41161 ctcatcgcta acagaacgca ccacttccca catgttttcc ggtgaccagc agcaccagac
41221 atgggcgcca gtgaagtaat ggcatttcca cgcatcggca taatcagatg ccaggtaaat
41281 gaactttccg ttgtcttctc cgattggact tatcgctccg cgcgtgagct ttccgttgat
41341 agctgagtct ttgttgtatc cagcacggaa aaacgttctg aataaatcac catccatgcc
41401 aatgaatgcc acgttgcacg gcttcattgt gtcagcgcgc atcatctcaa ccgcaataag
41461 ctctccagat ttacctctgg tgacattaac gtcgcaacca ataccattta tcaatgtcca
41521 ctcattcatc gcgacacgcg tcgttttggc catgattgct gcgacttcct gctcggtgat
41581 gaagttatcg ttgtaattta tcttcggcaa ctccttccta attgcctcca gcttttcacg
41641 cgggtgcata ttcaggaatc caccaagtgc ctccagtgct tcgggaaagc tcatgcctga
41701 aagtttcatc agccagttca tgccgctacc ggaaccgcac tgattgcata ttgcgccgcc
41761 gtcgcctttt gtttcgaagt tgtcatcgaa gcgatacctg tcttttcctg agcatgacgg
41821 gcatggctga tgcttgccat tgaaaactct gctatccaca ttgacgatag acatgatggc
41881 ggcttgccag ttgccaagca tttttggctc aatgtctttc cagtagtatc tcataaaatc

41941 ttcctgttga ctgtgatgta gaatcattgt aatttactca catgaaacaa tcaatcgtta
42001 cttgcaatga aacaattaat cgttacaata gaggataaga aatggctatc accgttaaga
42061 agtgcgaggt ttgcggaaat gagtttatcg gaactgcaaa agcaaaatgt tgctcaggaa
42121 agtgcagatt gcgcaagcac agacagaaga aaaacccaat tcataactca aaaacagata
42181 atggaatatc ttgaatataa ccctgagact ggtgttttta cagctgcaaa aacacatgga
42241 acactctggc gtaaaggcaa gattgttggg cataagaata aggcagggta catcaccatc
42301 acgctactgg gaaaactaag gaaagctcac aggctggcat ggatttatgt ttatggagaa
42361 gatatagatg ggtatgaaat agaccatata aatggtgaca aatcagacaa tagaatttgc
42421 aaccttagga tttctagtca ccaacaaaac atgttcaaca tgaaaaagaa atcaacaaat
42481 aaatctggtg taaaaggtgt gcatttcgat aaagggtgca acaaatggag agcgcagaca
42541 tcaataaaca agaagagggt tcatcttggt ttgtttgaca ccatagaatc agcagaaaag
42601 gcgattcgtg aattcatggt tgctaaccat aaagagttta ttaatttagg gtgagttatg
42661 cataagatag acaaaatgat ttcagagatt gacattaact tgctgaaatc ctgcttggat
42721 actggtgata tcgagccaag accatatcag tggttgatat ataagttaac tggtgatgtg
42781 atccgccatt atgttggtcc aagctatgta acagcaagcg tcggtagcgg aaagtcactg
42841 atgattgcca tgatagcaaa gcgttttcag gaaatgggat attcagggat gattttatca
42901 agacagggcg aaatagtgga gcaggatgcg gaagagctat ggtcgtttgg tgttagaaac
42961 tcgctattca gtgcttcgtt aggtaggaaa agttctacgt acccaataat ctgtggttct
43021 gagggtactg ttgtaaatgc tttgtttgat aaaaaggatg ggtcagggaa tgttattgca
43081 aaaggtgtgc tctctgactt ttgcccaaga ttcctgctga tcgatgaaaa tcatatggtt
43141 aatgacattg atgttgttaa taatggtgat acgcagtatg cagtgataat taatgagcta
43201 atgaaaaggt gcaaagataa gcacggccat gagttgagaa taataggtta tacaggctca
43261 ccgtttcgcg gaactacatc aataaaaggc gctttctgga agaaagagat tatcaacata
43321 gacaccaagt acatggttga aaacggtttt cttgtaccaa caatttttgg tcttcatgat
43381 gttgatagtc tgcattatga cctttcagat tttcatggtt cagatgttga tggtactcag
43441 gactttaccg ccgagcagct caagcagatg cagaaagaaa ttcttgagca aggtacgttg
43501 acgcagaaaa tcatgctcaa ggttatggaa ttgactaaaa acaggaacgg agtgctaatt
43561 acatgtgctg gtaaaaagca ttgccaggag gcggcaaagt atttgcctga aggaagttat
43621 tcaatagtta cagaagatat ggggtcaaaa gccagaagga aggctctaaa agatgcatac
43681 accgggcgca aaaaattcac attccagata gctgccctta ccactggcgt aaatataccg
43741 ttatgggata cgagctgcat attgcgaaaa ataatgtcac tcactcttct tgttcagttg
43801 cttggtcgcg ggatgcgcct gctgaagaaa gagcaaattg atgccgggta tcataaagag
43861 gaccatctgg ttcttgattt ctctggcacg atgtttgagc ttggtcagct atatgaagac
43921 ccaatccttg aagaggctga agcgcaacgt tcaaaacgca gtggtgaaca agttccgtgc
43981 ccgaaatgcg gaacaatgaa cagcccatat gcgcgccgct gcattggcaa agatgcattg
44041 tcgccagatg gtagatgcga agagtttttc agttatatcc gttgcggttt cgacaaacat
44101 ggcatccgta tttttgatga tggatgcggc actaaaaacg accccactgc gcgttattgc
44161 cgacattgcg atcatgtttt gcgcgacccg aatgcggcac ttaatgagcg tgcgtataca
44221 gataatgagt gggctgatgt tatggatttc aaagtccagt taacgaagga tggagaaggt
44281 attttgtatc gctactggat taatcgttgt gatggcaaag aaggctgggc taacgaagtt
44341 ttctaccctt atggcggcgc aactcacatg aaaaacatgt ttaaggcaaa ggcggtcttt
44401 cctcaccttg atgataagtc aatggcgggg aagatcctga aatgccagaa cgccaagcaa
44461 ttcatgatgt atgcgggatt gattaaagcg ccaaaacgca tcacgcatcg aattaatgat
44521 aaaggtcgcg acataataca ccgcaaagaa tttaaagggg aacaaagtga agcagcttga

44581 tagcggtata tgggtatttg atagcggtta tcgcggagaa tgcccaaaag aggagatcga
44641 ccagatgggt tacggtacat ggatgcaaca ccgtttccct gatgttctgt ggtttcacgt
44701 cccgaatgaa acaggaacat ccagccgcgt ccagttcgta ctgaagcgcc agaagatggg
44761 tgttaagact ggaataggcg acaatgtgat aatgacgcct ggagtgaagc acacttgcgg
44821 aatgattgag gcaaagcgcc gcgataaaag caaaagcaga gtgagcaagg agcaatctac
44881 tgtattaact gagatgtgca ggcttggtca ttatgccgca atagcttatg ggcttgatga
44941 attaaaaaaa gctactttat tctattttgg acttgatgaa tgacgtagat tgatgtagat
45001 tcaattacac aatgacaagt gaggtgatga agatggatat tgagattccg gatagttttg
45061 atgctgagtg gcaatgcgaa atgctacgca atcttcttgt taagttaaac gaacttgatg
45121 atggaggtta tgttgtcagc gatggttatt cattgcttga tgatgcaatg aaaattgtcg
45181 aagcgttacg tgaatacagt ggtgattaaa tgaaagtcta tttcaataat gaattaacta
45241 atgagcaata ccacgctgac acagagcaca tcaacggttc tggcctgtgg aacatttatg
45301 accgctgccc cgcagcttgg cgctacaaag acgaagaaga tgagcagtca aaagctctta
45361 tcttcggaac cggaagccat acggctctac ttgaacctga gcgctttgat gcggaatatg
45421 ctcgcatgcc aaccaaagaa gattttggtg atgacctgct tgttactgta agcgatatga
45481 actcatgggc gaaagagcgc ggcattaaag gactatcagg gaagtcgaaa gctgaggtga
45541 tcaaaataat tcgtgcaact ggcgaaccag tgaagattta cgacgaagag cgtcttattg
45601 ctgaaattaa cgccaatggt cgcactctgt tagaaggtga tgattatgac gtcattcagc
45661 aaatgcgtgc tgtaattcac gcaaacagct attacagcag ccttcttgct ggttcttatg
45721 ctgaagtatc aatcctcggt gagctttacg gggaaaaggc aaaggttcgg tttgactgcc
45781 tgacaaaagg tggtgacata attgactaca agacagcggt aagcgccaag cctgatgagt
45841 ttttccgtca tgctgcgcga cttggctact ttatgaagat ggcgatgcag cacgacatgt
45901 ttgttgcggc atacggtcat gcgccgcgtt ctgtaaacct tttagttcag gaaaagaaag
45961 cgccgtttat tcctgcgtta attctcttga ctgaagagca attacgcatc ggtcgcattc
46021 agttgaatgg tgcaatggaa atttacaagg cgtgtaaaaa agccaattca tggccgggtt
46081 attcaatggg taatcctgtt atcgaaatgg aaacgcctga gtggttcaag aaacagttta
46141 atttataatt aatgaggtga tgcaaatggg tattctcaat atcaaaccag cagagcgttc
46201 aggttctcgt gtggtcatcg gtatttcagg tcagtctggt agtggtaaaa catatagtgc
46261 acttaagctg gcgcgaggta tggttgattc accagaggaa atcggattcc ttgatactga
46321 aaacggtcgc ggtcgcctgt actcaaacat ccttgatggt aaattcctgc acgctgatat
46381 gtatgctcca ttcagcccgg ctcgctaccg tcaggccatt gaagagtttc aggttgctgg
46441 cgttaaggtt cttgttattg attctggctc gcacgaatgg gaaggggaag gaagttgcac
46501 ggagattgct gaaaagcctc ttcttaacgg taaaaagatg gctgactgga agcgcgccaa
46561 agctgaacac aagaagttca tgaacgccat gctacaaagc aatatgcata tcattgtctg
46621 tcttcgcgct cgccagaaaa ctgacttcgc aaacccgaag gagcctgtat cacttgggtt
46681 gcagccagtg tgcgagaaag atttcatgtt cgagatgacc gtgagcatga tgatgcacga
46741 tggaggcaaa attcaggagt tcaccaaact accagaagag ctacgtccaa tcttctttga
46801 gtctggtcgt gaaagtgttc gtcatggata cattggcgag gctcacggtc gcggactcat
46861 caaatgggtg gactctggcg ttaaagttga cgaagaattt gagtcatggc gctcacgact
46921 tcagctttcc gccgcaaaag gcatggaagg cctgaaggaa gaagcaaaat caatacctga
46981 taacctgaaa gataaaattc gagcaatctg gccttctctg gctgcttcgg ccgcagaata
47041 tgatcgcatt gagtctttca tcaatgatga gcaattatcc cctgtggtaa taactcctca
47101 ggataatttc aaccctgcaa aactggccaa acagcaacca cagcagccag aacagcagga
47161 agaaacaaaa accgaacata aaccaactcc aattgaagga ttttaataaa tggctcgcgg

47221 cgtaaataaa gtaattattg ttggcactct cggaaacgac cctgaagtta aatattcagc
47281 atcaggctct gcaattgcca acctttctgt tgcaacatct gatcagtgga aagacaagca
47341 gacaggagaa aagaaagagc agactgaatg gcatcgcgtg gtcatctttg gaaaacttgc
47401 agaagttgcc ggagaatatc ttcgcaaagg ttcacaggtt tacatcgaag gccagcttcg
47461 aactcgcaag tggactgaca gcaacggtgt ggacaaatac accactgaga ttgtcattcc
47521 acagatgggc ggagttatgc agatgcttgg aggtaaacgt gatgattctg gtcagcaaca
47581 accacgccag cagtcagggc aacaaccgca a
